# *Pseudomonas* Cyclic Lipopeptides Suppress the Rice Blast Fungus *Magnaporthe oryzae* by Induced Resistance and Direct Antagonism

**DOI:** 10.3389/fpls.2019.00901

**Published:** 2019-07-10

**Authors:** Olumide Owolabi Omoboye, Feyisara Eyiwumi Oni, Humaira Batool, Henok Zimene Yimer, René De Mot, Monica Höfte

**Affiliations:** ^1^Laboratory of Phytopathology, Department of Plants and Crops, Faculty of Bioscience Engineering, Ghent University, Ghent, Belgium; ^2^Centre of Microbial and Plant Genetics, Faculty of Bioscience Engineering, KU Leuven, Heverlee, Belgium

**Keywords:** lokisin, anikasin, WLIP, entolysin, xantholysin, CLP N3, non-ribosomal peptide synthetase, ISR

## Abstract

Beneficial *Pseudomonas* spp. produce an array of antimicrobial secondary metabolites such as cyclic lipopeptides (CLPs). We investigated the capacity of CLP-producing *Pseudomonas* strains and their crude CLP extracts to control rice blast caused by *Magnaporthe oryzae*, both in a direct manner and via induced systemic resistance (ISR). *In planta* biocontrol assays showed that lokisin-, white line inducing principle (WLIP)-, entolysin- and N3-producing strains successfully induced resistance to *M. oryzae* VT5M1. Furthermore, crude extracts of lokisin, WLIP and entolysin gave similar ISR results when tested *in planta*. In contrast, a xantholysin-producing strain and crude extracts of N3, xantholysin and orfamide did not induce resistance against the rice blast disease. The role of WLIP in triggering ISR was further confirmed by using WLIP-deficient mutants. The severity of rice blast disease was significantly reduced when *M. oryzae* spores were pre-treated with crude extracts of N3, lokisin, WLIP, entolysin or orfamide prior to inoculation. *In vitro* microscopic assays further revealed the capacity of crude N3, lokisin, WLIP, entolysin, xantholysin and orfamide to significantly inhibit appressoria formation by *M. oryzae*. In addition, the lokisin and WLIP biosynthetic gene clusters in the producing strains are described. In short, our study demonstrates the biological activity of structurally diverse CLPs in the control of the rice blast disease caused by *M. oryzae*. Furthermore, we provide insight into the non-ribosomal peptide synthetase genes encoding the WLIP and lokisin biosynthetic machineries.

## Introduction

Fluorescent *Pseudomonas* spp. belonging to the phylum Proteobacteria are prominent among rhizosphere and soil microbes (Philippot et al., 2013). The biocontrol capacities of rhizosphere-associated *Pseudomonas* species have been extensively studied (Haas and Défago, 2005; Weller, 2007; D’aes et al., 2010; Höfte and Altier, 2010; Olorunleke et al., 2015b; Stringlis et al., 2018). *Pseudomonas* species are metabolically versatile and produce an array of secondary metabolites including various antibiotics and cyclic lipopeptides (CLPs) (Gross and Loper, 2009). CLPs are amphiphilic molecules made up of a cyclic oligopeptide lactone ring linked to a fatty acid tail (Raaijmakers et al., 2006, 2010). CLPs are synthesized by non-ribosomal peptide synthetases (NRPSs) which encompass different modules containing condensation, adenylation and thiolation domains for co-linear synthesis of a specific (lipo)peptide and its release in linear or cyclic form by thioesterase activity (Finking and Marahiel, 2004; Strieker et al., 2010). *Pseudomonas* CLPs are currently classified into at least 14 different groups based on the length of the oligopeptide (Gross and Loper, 2009; Olorunleke et al., 2015b; Geudens and Martins, 2018). The lipid moiety in these groups is usually a hydroxydecanoic acid. Under *in vitro* and *in vivo* conditions, *Pseudomonas* CLPs have been shown to possess biocontrol potential against several plant pathogens including *Pythium myriotylum* (Oni et al., 2019a,b), *Pythium aphanidermatum* (Michelsen et al., 2015), *Phytophthora infestans*, *Phytophthora capsici* and *Pythium ultimum* (Van Der Voort et al., 2015), *Rhizoctonia solani* (D’aes et al., 2014; Michelsen et al., 2015; Olorunleke et al., 2015a), *Cochliobolus miyabeanus*, and *Magnaporthe oryzae* (Ma et al., 2017).

Biocontrol of plant pathogens using CLPs may involve direct antagonism and/or indirect activity via induced systemic resistance (ISR). ISR can occur in plants by the perception of elicitors secreted by microbes (or pathogens), similar to the microbe (or pathogen)-associated molecular patterns (MAMPs or PAMPs) (Newman et al., 2013). MAMPs or PAMPs are recognized by pattern recognition receptors associated with the cell membrane resulting in the elicitation of plant innate immunity (Boutrot and Zipfel, 2017). Diverse elicitors secreted by plant beneficial bacteria can trigger ISR (De Vleesschauwer and Höfte, 2009; Mariutto and Ongena, 2015), including some CLPs produced by *Pseudomonas* spp. (massetolide A, orfamide and sessilin) (Tran et al., 2007; Ma et al., 2016b, 2017), and by *Bacillus* spp. (surfactin, fengycin, and iturin) (Ongena et al., 2007; Jourdan et al., 2009; Henry et al., 2011). There are no indications, however, that plants perceive CLPs by specific receptors. Results with the *Bacillus* CLP surfactin indicate that perception relies on a lipid-driven process at the plasma membrane level (Henry et al., 2011).

Rice (*Oryza sativa L*.) is a major staple for more than 50% of the world population and is an essential food crop globally (Fageria and Baligar, 2003). However, this crop is affected by both abiotic and biotic stresses (Mittler, 2006; Pandey et al., 2015). Of the biotic stresses common to rice, the blast disease caused by the filamentous ascomycete, *M. oryzae*, has been ranked among one of the most important diseases of rice due to its widespread occurrence and destructiveness (Dean et al., 2005). This fungus is a hemibiotroph such that the infection process requires an initial biotrophic phase wherein the pathogen forms bulbous invasive hyphae within healthy plant cells (Koga, 1994). This is followed by a switch to necrotrophic growth, resulting in the death of plant cells. *M. oryzae* utilizes appressoria to penetrate the cuticle of rice by means of turgor pressure generation (Wilson and Talbot, 2009; Yan and Talbot, 2016).

Biological control of *M. oryzae* due to beneficial *Pseudomonas* species strains has been scantily reported (Spence et al., 2014; Hernández-Rodríguez et al., 2018; Wu et al., 2018). Orfamide produced by beneficial *Pseudomonas* species was reported to induce systemic resistance in rice against the brown spot fungus *Cochliobolus miyabeanus* but not to *M. oryzae* VT5M1 (Ma et al., 2017). More so, orfamide-producing strain *Pseudomonas protegens* CHA0 did not induce resistance to *M. oryzae* in rice (Spence et al., 2014). Another study showed the direct effect of orfamide in the inhibition of appressoria formation by *M. oryzae* and reduction of the severity of blast on rice (Ma et al., 2016a). *Pseudomonas mosselii* BS011 showed strong inhibitory activity against *M. oryzae* and a gene cluster that most likely mediates xantholysin production was required for this activity. In addition, a crude extract of *P. mosselii* BS011 inhibited the development of *M. oryzae* and impaired appressoria formation (Wu et al., 2018).

In a previous study, we characterized CLP-producing *Pseudomonas* strains associated with the cocoyam rhizosphere in Cameroon (Oni et al., 2019a). Representative CLP-producing strains effectively suppressed the cocoyam root rot disease caused by *Pythium myriotylum*. CLPs described in our previous study include entolysin, lokisin, putisolvin, xantholysin, WLIP, cocoyamide A and seven novel ones (Oni et al., 2019a). Furthermore, we showed that purified lokisin, entolysin, xantholysin, and WLIP could interact with the mycelium of *P. myriotylum* resulting in hyphal leakage and/or branching (Oni et al., 2019a). This rich CLP collection provided us with an opportunity to investigate the possible role of CLPs other than orfamide and xantholysin in the biological control of rice blast caused by *M. oryzae* via ISR or direct antagonism.

## Materials and Methods

### Strains, Media and Growth Conditions

*Pseudomonas* and *M. oryzae* strains used in this study are presented in Table 1. *Pseudomonas* wild type strains were cultured on King’s B (KB) (King et al., 1954) agar at 28°C for 48 h. WLIP-deficient mutants of *P. putida* RW10S2 were grown on KB agar supplemented with 50 μg/ml kanamycin at 28°C. Broth culture of *Pseudomonas* strains were obtained in KB broth on a rotary shaker at 150 revolutions per minute (rpm) and incubated at 28°C for about 24 h. *M. oryzae* isolate VT5M1 (Thuan et al., 2006) was grown on complete medium (CM) (Talbot et al., 1993), at 28°C for 5–8 days.

**Table 1:** Strains used in this study.

Strain	Relevant characteristics	References
***Pseudomonas koreensis* group**		
COW3	N3-producer from cocoyam rhizosphere, Cameroon	Oni et al., 2019b
COR10	Lokisin producer from cocoyam rhizosphere, Cameroon	Oni et al., 2019b
***Pseudomonas putida* group**		
COR5	Entolysin-producer from cocoyam rhizosphere, Cameroon	Oni et al., 2019b
COW10	WLIP-producer from cocoyam rhizosphere, Cameroon	Oni et al., 2019b
COR51	Xantholysin producer from cocoyam rhizosphere, Cameroon	Oni et al., 2019b
NSE1	WLIP producer from the white cocoyam rhizosphere, South Eastern part of Nigeria	Geudens, 2017; Oni et al., unpublished
RW10S2	WLIP producer from the rice rhizosphere, Sri Lanka	Rokni-Zadeh et al., 2012
CMPG2170	*wlpA* (NRPS1) mutant of RW10S2	Rokni-Zadeh et al., 2012
CMPG2169	*wlpB* (NRPS2) mutant of RW10S2	Rokni-Zadeh et al., 2012
CMPG2120	*wlpC* (NRPS3) mutant of RW10S2	Rokni-Zadeh et al., 2012
BW11M1	Xantholysin producer from the banana rhizosphere, Sri Lanka	Li et al., 2013
***Magnaporthe oryzae***		
VT5M1	Rice blast pathogen from Vietnam	Thuan et al., 2006
Guy11	Rice blast pathogen from French Guyana	Leung et al., 1988

### Extraction of Crude CLPs

Extraction of crude CLPs from *Pseudomonas* strains was carried out following an established protocol (Oni et al., 2019a). Specifically, *Pseudomonas* strains were grown on KB agar overnight after which seed cultures were obtained by growing bacterial cells in 5 ml KB broth in sterile glass tubes, at 150 rpm and 28°C for 24 h. The 24 h old culture was subsequently transferred into 2 L Erlenmeyer flasks containing 400 ml KB broth and incubated at 150 rpm and 28°C for 24 h. Cell-free supernatants were obtained by centrifugation of the culture at 10,000 *g* and 4°C for 10 min followed by acidification to pH 2 using 6 N hydrochloric acid with constant stirring. Acidified culture supernatant was kept overnight at 4°C in order to precipitate the CLPs. Precipitated CLPs were collected by centrifugation at 10,000 *g* for 10 min after which crude CLPs were extracted using 100% methanol. The solvent was evaporated at room temperature to obtain crude CLP samples. Crude orfamide A was extracted from *Pseudomonas protegens* CHA0 following a different published procedure (Ma et al., 2016a).

### Plant Material and Growth Conditions

Rice (*Oryza sativa* L*.)* indica cv. CO-39 was used as plant material for all bioassays. Paddy rice seeds were dehusked and surface-sterilized with 2% (w/v) sodium hypochlorite solution by shaking on a rotary shaker for 10 min at 100 rpm, and room temperature. The seeds were rinsed several times with sterile distilled water, and air-dried. Air-dried seeds were germinated in the dark at 28°C on sterile filter paper in Petri dishes supplemented with 5 ml sterile distilled water. After 5 days, pre-germinated rice seeds were exposed to light at 30 ± 4°C for 2 days. Rice seedlings were transplanted into plastic trays (23 × 16 × 6 cm, 12 plants in each) using non-sterile potting soil (Structural; Snebbout, Kaprijke, Belgium) as substrate (De Vleesschauwer et al., 2006; Ma et al., 2016a, 2017). Rice plants were kept in a greenhouse (photoperiod with 12 h light at 30 ± 4°C). Watering was done every 3 days, and 200 ml of nutrient [FeSO_4_; 2 g/L and (NH_4_)_2_SO_4_; 1 g/L] was applied to each tray once a week.

### ISR Potential of *Pseudomonas* spp. Against *M. oryzae* VT5M1

For ISR assays in rice, *Pseudomonas* wild type strains were grown on KB agar at 28°C for 48 h, while WLIP-mutants were grown on KB agar supplemented with 50 μg/ml of kanamycin. Bacteria were scraped from plates and suspended in 0.85% sodium chloride (w/v). Bacterial density was determined by measuring the optical density (OD) at 620 nm. The inoculum of bacteria was calculated based on 600 g of soil and adjusted to a final density of 1 × 10^7^ CFU/g. Twenty five μg/ml of *S*-methyl 1,2,3-benzothiadiazole-7-carbothioate (BTH) (a kind gift from Syngenta Crop Protection, Brussels, Belgium) was used as the positive control. Bacterial suspensions and BTH were diluted with 250 ml of water and mixed with 600 g of potting soil for 5 min. Roots of rice seedlings obtained as mentioned earlier were inoculated with the standardized bacterial inoculum or BTH by soaking for 10 min before planting them in plastic trays containing potting soil mixed with the respective bacterial suspension or BTH. For each strain and the BTH control, three biological replicates of 12 plants were used. Bacteria and BTH were added as soil drench 3 days before inoculating 4 weeks-old rice plants with *M. oryzae* VT5M1. Spores of 5-days old *M. oryzae* VT5M1 were suspended in 0.5% (w/v) gelatin to final concentration 5 × 10^4^ spores/ml. Rice plants were evenly sprayed with 1 ml of spore suspension using a compressor-powered airbrush gun (Badger Airbrush model 150^TM^). Inoculated rice plants were kept for 22 h in a dark chamber (relative humidity ≥ 90%; 25 ± 5°C), and further transferred to the greenhouse for disease development. Disease was scored 6 days after infection by counting the number of susceptible lesions (ellipsoid to round-shaped lesion with a gray center indicative of sporulation) shown by the second stage youngest leaves (De Vleesschauwer et al., 2006; Ma et al., 2016a). Pictures of typical disease symptoms were taken 7 days after infection. The bioassay was carried out twice.

### Root Colonization Assay

Colonization of rice roots was evaluated as published previously (Ma et al., 2017; Oni et al., 2019a). Specifically, roots of five rice plants from each treatment were randomly chosen after disease evaluation, and rinsed carefully with water to remove soil. The roots were cut into small pieces and weighed. Bacterial suspension was obtained by crushing the roots using a mortar and pestle in 10 ml sterile saline (0.85% sodium chloride, w/v) and fine sand. Exactly 100 μl of the serially diluted suspension was plated on KB agar and incubated for 24 h at 28°C. Fluorescent *Pseudomonas* bacterial colonies were counted after 24 h and the data were log10 transformed prior to statistical analysis.

### Induction of Systemic Resistance to Rice Blast by Crude Pseudomonas CLPs

Crude CLPs extracts were dissolved in 250 ml of distilled water to obtain a final concentration of 25 μg/ml. Twenty five μg/ml of BTH was used as positive control. Crude CLPs and BTH were separately mixed with potting soil for 5 min. Rice seedlings obtained as earlier described, were soaked in 25 μg/ml of each CLP/BTH for 10 min and planted in plastic trays (containing 600 g of soil mixed with crude CLPs, three biological replicates of 12 plants per tray). A second application of 25 μg/ml of each crude CLP or BTH was conducted using 250 ml of tap water as soil drench 3 days before inoculation of the rice plants with *M. oryzae* VT5M1. Preparation of spore suspension, inoculation of the rice plants, and disease evaluation were done as described above. The bioassay was conducted twice.

### Co-application of CLP-*M. oryzae* Spore Mixtures on Rice Leaves

Pre-germinated rice seedlings were grown in plastic trays containing 600 g of non-sterile potting soil as mentioned above, and maintained under greenhouse condition for 4 weeks. Spore suspensions of *M. oryzae* VT5M1 were prepared using the modified method described previously (Thuan et al., 2006). Specifically, cultures of *M. oryzae* VT5M1 were grown for 8 days at 28°C. Spores were collected and suspended in 0.5% (w/v) gelatin, mixed with crude CLPs to final concentration of 5 × 10^4^ spores/ml, and 25 μg/ml of each CLP. The mixture was evenly sprayed on rice plants using a compressor-powered airbrush gun, while the trays were frequently rotated during this procedure. Each plant received 1 ml of the mixture. Healthy control plants received only 0.5% gelatin suspension, positive control plants were sprayed with a mixture of 0.5% gelatin, 5 × 10^4^ spores/ml and 25 μg/ml of BTH, while diseased control plants received 1 ml 5 × 10^4^ spores/ml and 0.5% gelatin mixture. Preparation of spore suspension, inoculation of the rice plants, and disease evaluation were done as described above. The bioassay was conducted twice.

### *In vitro* Bioassay With *M. oryzae* Isolate VT5M1 and Crude CLPs

Different crude CLPs used in this study were dissolved in 100% dimethylsulfoxide (DMSO) as stocks. Further dilutions were made in 0.1% DMSO to get the desired concentrations for bioassays. Five-day old *M. oryzae* isolate VT5M1 spores (5 × 10^4^ spores/ml) were collected from CM agar plates and mixed with different crude CLP extracts and 0.1% DMSO was used as a control. In this experiment, 100 μl of different concentrations: 1, 5, 10, and 25 μg/ml of each crude CLP was mixed with 100 μl of *M. oryzae* while the negative control received 100 μl of 0.1% DMSO. Fifty μl of the mixture was transferred onto a plastic slide (Fisher Scientific, Belgium) and incubated at 28°C. Spore germination was evaluated by counting the number of germination tubes after a 4 h incubation period. Appressoria formation was assessed by randomly counting at least 50 spores after 8 h incubation. Microscopic observations were carried out using an Olympus BX51 Microscope. The experiment was carried out twice.

### *In vitro* Antagonism of Xantholysin-Producing Strains Against *M. oryzae*

A Petri dish assay was used to investigate the *in vitro* antagonism of the xantholysin-producing strains COR51 and BW11M1 against *M. oryzae* VT5M1 and *M. oryzae* Guy11, as described by Desai et al. (2002) and Wu et al. (2018). Specifically, 3 μl of overnight Luria Bertani (LB) broth culture of the test bacteria were spotted on either side of CM plates spanning 2 cm from the center. The plates were incubated for 24 h at 25°C, after which a mycelium plug of VT5M1 or Guy11 (5 mm in diameter) was placed at the center of the plate. Plates were re-incubated at 25°C for 6–10 days.

### Draft Genome Sequencing of CLP-Producing *Pseudomonas* Strains

Genomic DNA was obtained from 24 h old culture of the lokisin-producing *Pseudomonas* strains COR10 and the WLIP-producing strain NSE1 grown in LB broth using the Wizard Genomic DNA Purification Kit from Promega (Promega Corporation, 2800 Woods Hollow Road Madison, WI, United States). Genomes were sequenced using the Illumina Hiseq 2500 or Miseq system platform at BaseClear (BaseClear B.V., Leiden, Netherlands). Paired-end sequence reads of the genomic DNA was generated using the Illumina HiSeq2500 system. FASTQ sequence files were generated using the Illumina Casava pipeline version 1.8.3. Initial and second quality assessments was based on data passing the Illumina Chastity filtering and using FASTQC quality control tool version 0.10.0 respectively. *De novo* assembly was performed using the CLC Genomics Workbench version 8.5.1. Mis-assemblies and nucleotide disagreement between the Illumina data and the contig sequences was corrected with Pilon version 1.11 (Walker et al., 2014). The scaffolding analysis was done using the SSPACE Premium scaffolder version 2.3 (Boetzer et al., 2011). Automated gap closure analysis was done using GapFiller version 1.10 (Boetzer and Pirovano, 2012).

### Genome Mining and Bioinformatics Analyses

For automated genome annotation and mining, genomic sequences of the *Pseudomonas* sp. COR10 and NSE1, together with the genomes of *Pseudomonas* strains listed in Supplementary Table S1 and extracted from the Genbank database, were submitted to RAST Server (Aziz et al., 2008) and antiSMASH 4.0 (Blin et al., 2017). Genome mining was conducted on the annotated genomes, and comparison of NRPS proteins with other protein sequences in GenBank database was done by BLAST search^1^. The adenylation (A) and condensation (C) domains sequences of the non-ribosomal peptide synthase (NRPS) genes were extracted. Sequence alignment was carried out using MUSCLE (Edgar, 2004) in the software package Geneious Prime, 2019, and the cladograms were inferred by Neighbor Joining^2^. The amino acids (AA) sequence generated by the biosynthetic gene clusters were predicted by submitting the A domains to NRPSpredictor2 database (Röttig et al., 2011).

### Phylogenetic Analyses of *Pseudomonas* spp.

The *rpoB* and *rpoD* sequences of the lokisin-, WLIP- and xantholysin-producing *Pseudomonas* strains used in this study were extracted from the draft genome sequences of the strains. Sequences of selected *Pseudomonas* type strains were retrieved from the GenBank (Supplementary Table S1). The sequences were aligned using MUSCLE (Edgar, 2004) in MEGA6 (Tamura et al., 2013). A phylogeny tree was constructed by Maximum likelihood with 1000 bootstrap replication, and *P. aeruginosa* was used as outgroup. *rpoB* and *rpoD* trees were generated separately after which a concatenated tree was obtained by combining the aligned sequences of the two genes.

### Statistical Data Analysis

SPSS 25 statistical software was employed for data analysis. To compare across treatments, univariate ANOVA followed by Tukey’s *post hoc* tests were used and results were considered to be statistically different when *p* < 0.05.

## Results

### Potential of CLP-Producing *Pseudomonas* Strains to Induce Systemic Resistance Against *M. oryzae* on Rice

**Figure 1:**
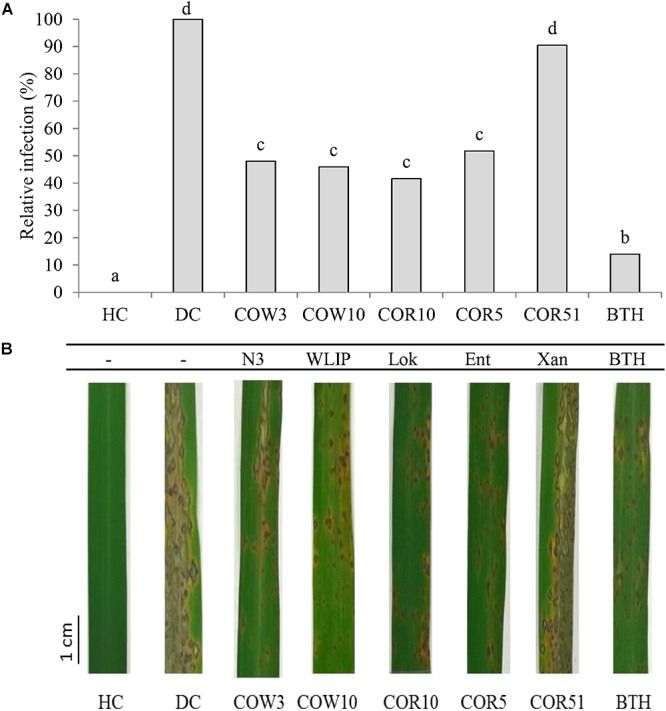
Induced systemic resistance (ISR) capacity of CLP-producing *Pseudomonas* spp. to *M. oryzae* in rice. **(A)** The potential of root application with *Pseudomonas* sp. strains COW3 (N3), COW10 (WLIP), COR10 (lokisin), COR5 (entolysin) and COR51 (xantholysin) to induce systemic resistance (ISR) against *M. oryzae* isolate VT5M1 on rice. HC: healthy control and DC: disease control plants were treated with water. Data of one experiment made up of three biological replicates, each consisting of twelve (12) plants are shown. The experiment was repeated with similar results. Different letters indicated statistically significant differences among different treatments (ANOVA followed by a Tukey’s tests: *n* = 36; α = 0.05), Lok, lokisin; Ent, entolysin; Xan, xantholysin; BTH, *S*-methyl 1,2,3-benzothiadiazole-7-carbothioate. **(B)** Representative pictures of disease symptoms observed on the second youngest leaf after 6 days post-inoculation with *M. oryzae*. Scale bar represents 1 cm.

The capacity of CLP-producing *Pseudomonas* strains to induce resistance against *M. oryzae* in rice was investigated by soil inoculation. Results showed that N3-, WLIP-, lokisin- and entolysin-producing strains, COW3, COW10, COR10 and COR5, respectively, successfully induced resistance to *M. oryzae* in rice. These CLPs-producing strains gave an intermediate level of protection of 52, 54, 58, and 48% for COW3, COW10, COR10 and COR5, respectively, in comparison to 86% reduction in disease severity for the positive control (BTH). However, the xantholysin-producing isolate, COR51 only reduced disease severity with 10%, and could not significantly protect the rice plants against *M. oryzae* (Figure 1A,B). Furthermore, rice roots were successfully colonized by *Pseudomonas* isolates tested in the range of 10^8^ to 10^9^ CFU/g fresh roots (Table 2).

**Table 2:** Population of CLP-producing *Pseudomonas* strains used in induced resistance assays on rice roots.

*Pseudomonas* sp.	CLPs produced	Colonization (Log CFU/g fresh roots)^∗^
COW3	N3	8.38 ± 0.38^*a*^
COW10	WLIP	8.63 ± 0.22^*ab*^
COR10	Lokisin	8.92 ± 0.32^*c*^
COR5	Entolysin	8.50 ± 0.43^*ab*^
COR51	Xantholysin	8.46 ± 0.35^*b*^

*No Pseudomonas bacteria were detected in uninoculated control treatments. ^∗^Values (±SD) followed by the same letter are not significantly different (Tukey’s test, α = 0.05)*.

### Potential of Crude CLP Extracts to Induce Systemic Resistance to *M. oryzae* in Rice

**Figure 2:**
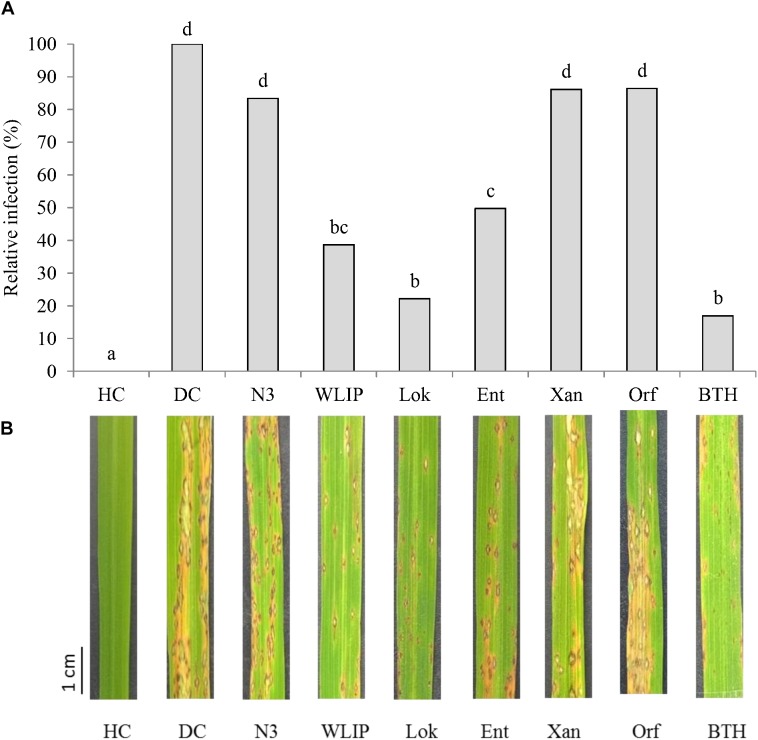
Induce systemic resistance capacity of crude CLP extracts against rice blast caused by *M. oryzae*. **(A)** ISR potential of crude WLIP, lokisin, entolysin, orfamide, and xantholysin to *M. oryzae* on rice. For each treatment, potting soil was mixed with 25 μg/ml of each CLP prior to experimental set-up. Subsequently, roots of rice seedlings were dipped in 25 μg/ml solution of each CLP for 10 min before planting. Data of one experiment made up of three biological replicates, consisting of 12 plants each are shown. The experiment was repeated with comparable results. Different letters indicate statistically significant differences among treatments (ANOVA followed by a Tukey’s test: *n* = 36; α = 0.05). **(B)** Representative disease symptoms on second youngest leaf at 6 days post-inoculation with *M. oryzae*. Scale bar represents 1 cm. DC, disease control; HC, healthy control; Lok, lokisin; Ent, entolysin; Xan, xantholysin; Orf, orfamide; BTH, *S*-methyl 1,2,3-benzothiadiazole-7-carbothioate.

The capacity of 25 μg/ml of crude extracts of N3, WLIP, lokisin, entolysin and xantholysin to induce resistance to *M. oryzae* VT5M1 on rice plants was tested in soil drench bioassays. Soil drenched with 25 μg/ml BTH was used as a positive control. Orfamide was used as a negative control since a previous study showed that this CLP is unable to induce resistance to rice blast (Ma et al., 2017). Lokisin, WLIP, and entolysin exhibited ISR against *M. oryzae* in rice plants. Lokisin gave a level of protection of 78%, followed by WLIP (61%), and entolysin (50%). In contrast, both xantholysin and orfamide reduced disease severity with 14%, while N3 reduced disease severity with 17%. These values were not significantly different from the diseased control (Figure 2A,B).

### Role of WLIP in Induced Systemic Resistance Against *M. oryzae* in Rice

**Figure 3:**
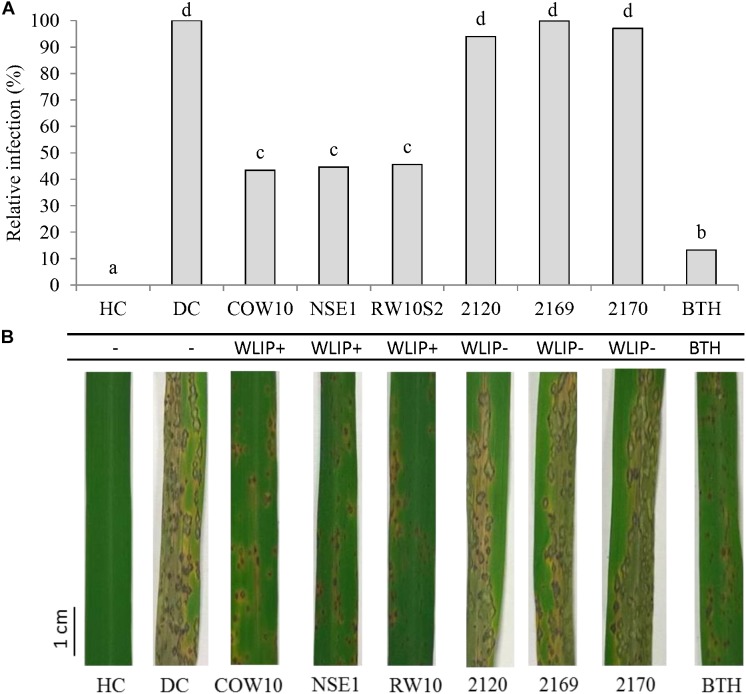
Role of WLIP in ISR against *M. oryzae* in rice. **(A)** Potential of WLIP-producing strain *P. putida* RW10S2, and biosynthetic mutants (CMPG2120, CMPG2169, and CMPG2170) in ISR against *M. oryzae* VT5M1 on rice. WLIP producers (COW10 and NSE1) and *S*-methyl 1,2,3-benzothiadiazole-7-carbothioate (BTH)-treated plants were used as positive controls. Data of one experiment made up of three biological replicates, consisting of 12 plants each are shown. The experiment was repeated with comparable results. Different letters indicate statistically significant differences among treatments (ANOVA followed by a Tukey’s test: *n* = 36; α = 0.05). **(B)** Representative disease symptoms on second youngest leaf at 6 days post-inoculation with *M. oryzae*. Scale bar represents 1 cm.

To further decipher the role of WLIP in ISR, we used the WLIP-producing *Pseudomonas* strains COW10, NSE1 and RW10S2 and previously generated WLIP-deficient mutants (*Pseudomonas putida* CMPG2120, CMPG2169, and CMPG2170) in the RW10S2 background (see Table 1) in a rice-*M. oryzae* bioassay. Results showed that WLIP-producing RW10S2 induces resistance to *M. oryzae* in a manner similar to other WLIP producers (COW10 and NSE1), but portrays an intermediate level of protection in comparison to the BTH control. However, WLIP-deficient mutants CMPG2120, CMPG2169, and CMPG2170 lost the capacity to induce resistance against *M. oryzae* (Figure 3A,B). The root colonizing capacity of all strains used attained 10^8^ CFU/g fresh roots (Table 3).

**Table 3:** Population of WLIP-producing *Pseudomonas* strains and mutants used in induced resistance assays on rice roots.

*Pseudomonas* spp.	CLPs produced	Colonization (LogCFU/g fresh roots)^∗^
COW10	WLIP	7.72 ± 0.07^*b*^
NSE1	WLIP	7.56 ± 0.39^*a*^
*P. putida* RW10S2	WLIP	7.62 ± 0.13^*ab*^
*P. putida* CMPG2120	–	7.69 ± 0.06^*ab*^
*P. putida* CMPG2169	–	7.67 ± 0.18^*ab*^
*P. putida* CMPG2170	–	7.74 ± 0.20^*b*^

*No Pseudomonas bacteria were detected in uninoculated control treatments. ^∗^Values (±SD) followed by the same letter are not significantly different (Tukey’s test, α = 0.05)*.

### Potential of Crude CLPs to Reduce Rice Blast Severity When Co-applied With *M. oryzae* Spores on Rice Leaves

**Figure 4:**
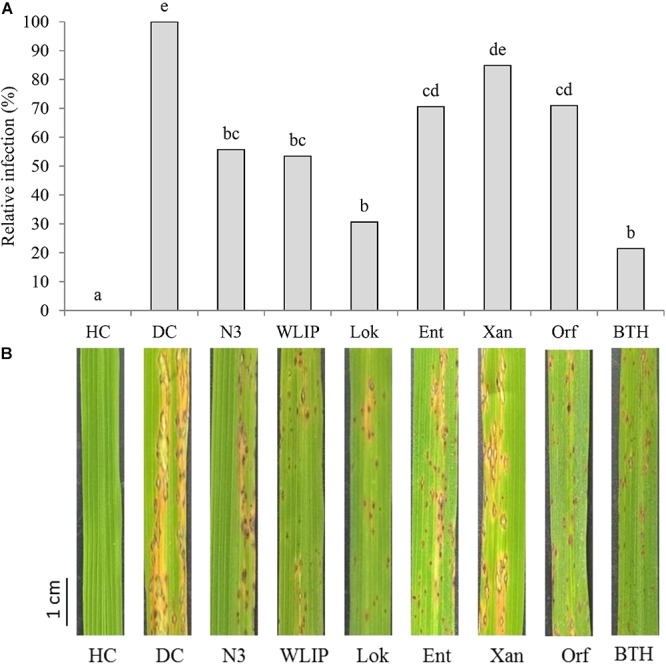
Effects of crude CLP extracts co-applied with *M. oryzae* spores on rice leaves on rice blast disease. **(A)** Effects of co-application of N3, WLIP, lokisin, entolysin, xantholysin, and orfamide extracts with fungal spores on rice blast caused by *M. oryzae*. Disease assessment was done at 6 days post-inoculation by counting the number of sporulating susceptible-type lesions occurring on the second youngest leaf of the rice plants. Data of one experiment made up of three biological replicates, consisting of 12 plants each are shown. The experiment was repeated with comparable results. Different letters indicate statistically significant differences among treatments (ANOVA followed by a Tukey’s tests: *n* = 36; α = 0.05). **(B)** Representative disease symptoms on second youngest leaf after 6 days post-inoculation with *M. oryzae*. Scale bar represents 1 cm. DC, disease control; HC, healthy control; Lok, lokisin; Ent, entolysin; Xan, xantholysin; Orf, orfamide; BTH, *S*-methyl 1,2,3-benzothiadiazole-7-carbothioate.

Crude CLP extracts were mixed with *M. oryzae* spores prior to fungal inoculation on rice leaves. N3, WLIP, lokisin, entolysin and orfamide extracts led to the reduction of blast disease severity on rice (Figure 4A,B). Relative reduction in blast severity ranged from 29% by entolysin and orfamide to 69% by lokisin, the latter providing protection comparable to BTH (79% reduction). Treatments with crude extracts of N3 (44%) and WLIP (47%) resulted in intermediate reduction in blast severity. However, the xantholysin extract reduced blast disease severity by only 15%, not significantly different from the diseased control.

### Effect of Crude CLP Extracts on *M. oryzae* Spore Germination and Appressoria Formation

**Figure 5:**
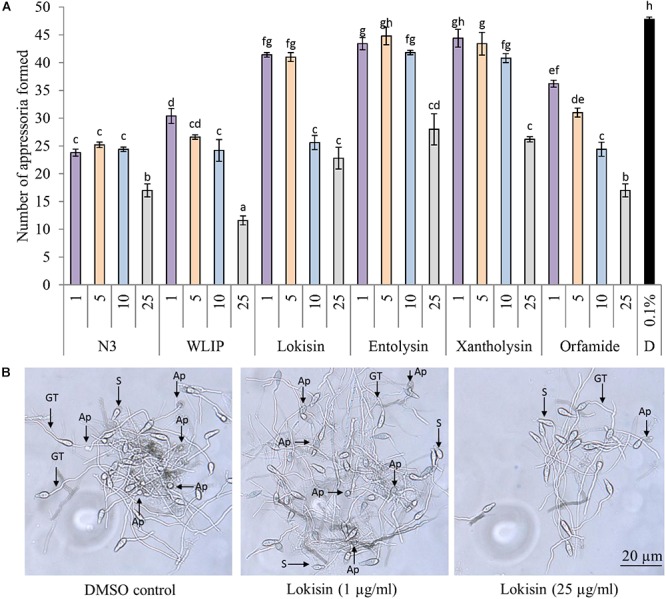
Direct effects of crude CLP extracts on appressoria formation in *M. oryzae.* **(A)** Direct effect of structurally diverse CLPs (1 – 25 μg/ml) on the formation of appressoria by *M. oryzae* VT5M1. Data shown are representative of two independent experiments. Different letters indicate statistically significant differences among the different treatments (ANOVA followed by a Tukey’s tests; α = 0.05), D: dimethyl sulfoxide (DMSO). **(B)** Representative pictures showing appressoria formation by *M. oryzae* VT5M1 in 0.1% DMSO and lokisin after 8 h of incubation. Ap, appressoria; GT, germ tube; S, spore. Scale bar represents 20 μm.

Under *in vitro* conditions, the direct effect of N3, WLIP, lokisin, entolysin, xantholysin, and orfamide extracts on *M. oryzae* VT5M1 spore germination and appressoria formation was investigated. Since orfamide was effective against the same pathogen during a previous study (Ma et al., 2016a), we included it in our study as a positive control. Orfamide and xantholysin extracts had no effect on the spore germination capacity of *M. oryzae* when compared to the DMSO control. Significant differences in spore germination relative to the DMSO control were observed for N3 (82%) and WLIP (70%) at 25 μg/ml, for lokisin at 5 μg/ml (80%) and for entolysin at 1 (80%) and 10 μg/ml (82%) (Supplementary Figure S1). However, all CLPs tested could inhibit appressoria formation at 10 and 25 μg/ml (Figure 5A). Relative appressoria formation is in the following order, WLIP (24%) > N3/orfamide (36%) > lokisin (48%) > xantholysin (55%) > entolysin (59%). Representative pictures showing the effect of crude lokisin on appressoria formation are shown in Figure 5B.

### *In vitro* Antagonism of Xantholysin-Producing Strains

In a previous study, crude xantholysin extract from *P. mosselii* BS011 significantly reduced blast severity when mixed with *M. oryzae* Guy11 and sprayed on rice plants (Wu et al., 2018). However, crude xantholysin from COR51 could not protect rice plants against *M. oryzae* following treatment with its spores-crude CLPs mixture. To further investigate the discrepancy with our results related to xantholysin and the report by Wu et al. (2018), we co-cultured *M. oryzae* VT5M1 and *M. oryzae* Guy11 with xantholysin-producing strains COR51 and BW11M1. *P. mosselii* BW11M1 is closely related to *P. mosselii* BS011 strain used by Wu et al. (2018), and the NRPS clusters encoding xantholysin of both strains are highly similar. *M. oryzae* Guy11 was more sensitive to COR51 than VT5M1, but the growth of both fungi was not completely inhibited. In contrast, BW11M1 strongly inhibited the growth of both fungi (Supplementary Figure S2). These observations warrant further investigation, but indicate that the strong antagonism caused by BW11M1 is probably not only due to xantholysin.

### Genome Mining for NRPS Genes Encoding Lokisin

**Figure 6:**
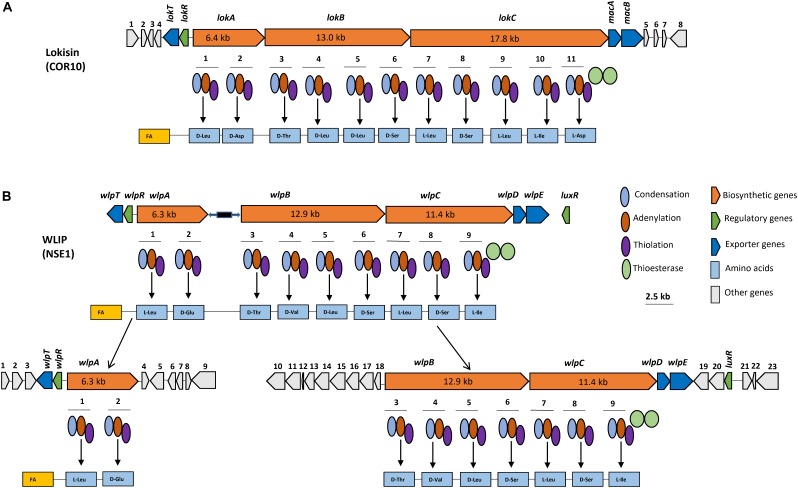
*In silico* analyses of NRPS gene clusters encoding WLIP and lokisin. **(A)** Gene clusters comprising three structural NRPS genes, designated *lokA, lokB*, and *lokC* responsible for lokisin biosynthesis in COR10. Detailed comparison of lokisin-synthetases and proteins encoded by the flanking regions are shown in Supplementary Table S3. **(B)** Gene cluster comprising three structural genes, designated *wlpA, wlpB*, and *wlpC* responsible for WLIP biosynthesis in NSE1. Detailed comparison of WLIP-synthetases and proteins encoded by the flanking regions are shown in Supplementary Table S4. Each module contains an adenylation (A), condensation (C) and thiolation (T) domains, and two TE domains for peptide release and cyclization. Scale bar represents 2.5 kb.

Genome mining carried out on the draft genome of the lokisin-producing *Pseudomonas* sp. strain COR10 showed the presence of three NRPS genes (Figure 6A). Integration of results obtained by submitting the NRPS gene cluster to the NRPSpredictor2, AntiSMASH 4.0, BLAST search and A domain analyses revealed the capacity to synthesize a CLP consisting of 11 AAs, with a peptide sequence identical to that of lokisin, produced by *Pseudomonas* sp. strain DSS41 (Sørensen et al., 2002) and anikasin produced by *P. fluorescens* HKI0770 (Götze et al., 2017) (Supplementary Table S2) and in agreement with the chemical structure elucidation reported for COR10 by Oni et al. (2019a). As the AA stereochemistry is not fully resolved for DSS41 lokisin (Sørensen et al., 2002) and not yet determined for COR10 lokisin, it can at present not be assessed unequivocally whether lokisin and anikasin (same lipopeptide sequence, absolute configuration determined; Götze et al., 2017) are different CLPs. The three NRPS genes of *Pseudomonas* sp. COR10 designated as *lokA*, *lokB*, and *lokC*, encode two, four, and five AAs respectively (Figure 6A). The two terminal thioesterase (TE) domains release the peptides from the NRPSs with concomitant cyclization. The NRPS genes are flanked by a *nodT*-like outer membrane lipoprotein gene, which we termed *lokT* and a *luxR*-type transcriptional regulator gene, which we termed *lokR*, upstream, and two transporter genes which are predicted to encode macrolide efflux proteins MacA and MacB, downstream. There is no *luxR*-type transcriptional regulator gene in the downstream region of the lokisin NRPS cluster (Figure 6A).

The genomic sequence of lokisin-producing strain *Pseudomonas* sp. strain DSS41 is not available in the GenBank database for comparative *in silico* analysis. However, BLASTp comparison showed a high identity of COR10 synthetases with arthrofactin synthetase of *Pseudomonas* sp. MIS38 (90–97%) and putative NRPS proteins of *Pseudomonas* sp. URIL14HWK12:I6 (97%), *P. koreensis* P19E3 (94–96%), *P. koreensis* BS3658 (86–93%), *P. koreensis* CRS05-R5 (86–92%), *Pseudomonas* sp. GM30 (90–96%), and *Pseudomonas* sp. Irchel s3a10 (90–97%). A somewhat lower identity was found with the anikasin synthetases of *P. fluorescens* HKI0770 (76–88%) and NRPS proteins in *P. chlororaphis* EA105 (77–83%), but the efflux and regulatory proteins in these last two strains showed a high identity with LokR (90%), MacA and Mac B (91%) of COR10 (Supplementary Table S3). NRPS A domains retrieved from these *Pseudomonas* strains were used to construct a phylogenetic tree, showing that strains URIL14HWK12:I6 and P19E3 are likely to produce lokisin. On the other hand, for strains GM30, BS3658, CRS05-R5 and Irchel s3a10 an isoleucine is predicted at the ninth position, similarly to the arthrofactin-producer MIS38, and they are probably also arthrofactin producers. The NRPS of EA105 would also recruit an isoleucine at position 9, but is predicted to incorporate a glutamine at the eighth position instead of the serine which is present in lokisin and arthrofactin, indicating that this strain may produce a novel member within the amphisin family (Supplementary Figure S3 and Supplementary Table S2).

The predicted D- or L- configuration of the AA residues of lokisin and other putative amphisin group CLPs based on C-domain phylogeny is shown (Supplementary Table S2 and Supplementary Figure S4). Although chemical validation is required to confirm the predicted stereochemistry, it can serve as a reliable clue to elucidate the configuration chemically. The lokisin biosynthetic gene cluster and flanking regions of *Pseudomonas* sp. COR10 have been deposited in Genbank with accession number MK534107.

### Genome Mining for NRPS Genes Encoding WLIP

Genome mining carried out on the draft genome of *Pseudomonas* sp. NSE1 showed three NRPS genes. Combined analyses by NRPSpredictor2, AntiSMASH 4.0, BLAST search and A domain analyses revealed synthetic capacity for a CLP comprising nine AAs, apparently identical to WLIP produced by *Pseudomonas putida* RW10S2 (Supplementary Table S5). WLIP synthetase of *Pseudomonas* sp. NSE1 is made up of three NRPS genes designated as *wlpA*, *wlpB*, and *wlpC*, which incorporate two, four, and three AAs respectively (Figure 6B). The biosynthetic gene cluster is flanked by a *nodT*-like outer membrane lipoprotein gene (*wlpT*) and a *luxR*-type transcriptional regulator gene (*wlpR)* upstream of *wlpA*, and two transporter genes (*wlpD* and *wlpE*) which encode putative macrolide efflux protein MacA and MacB downstream of *wlpC* (Figure 6B). Furthermore, BLASTp searches of the NRPS cluster of *Pseudomonas* sp. NSE1 revealed as high identity with the corresponding proteins in *Pseudomonas* sp. RW10S2 (94–96%), and an even higher identity (99%) with *Pseudomonas putida* NX-1 (Xu et al., 2018) and *Pseudomonas putida* PC2 (Song et al., 2017) (Supplementary Table S4). In line with this, A-domain phylogenetic analysis (Supplementary Figure S3) suggests that *Pseudomonas* spp. NSE1, NX-1 and PC2 are WLIP producers. The result showed the same AAs as the previously reported WLIP producer, RW10S2, were recruited by our strain, NSE1, together with NX-1 and PC2 strains (Supplementary Table S5 and Supplementary Figure S5). The predicted isomery (D and L configurations) of the AAs sequences of WLIP based on C-domain phylogeny are shown (Supplementary Table S5 and Supplementary Figure S6). The predicted chemical isomery could offer a clue to elucidating the chemical configuration for validation purpose. The WLIP biosynthetic gene cluster and flanking regions of *Pseudomonas* sp. NSE1 have been deposited in GenBank with accession numbers MK534106 and MK650230.

### Phylogenetic Analysis of *Pseudomonas* spp. Based on *rpoD* and *rpoB* Gene Sequences

**Figure 7:**
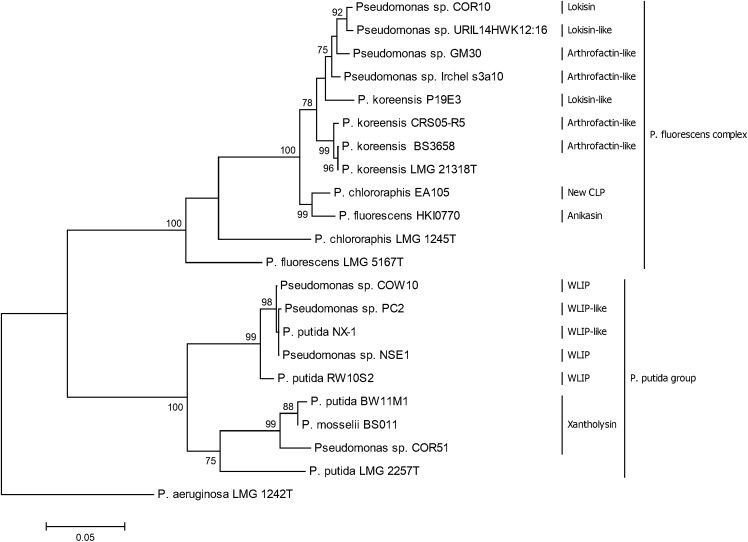
Phylogenetic analysis of *rpoD* and *rpoB* partial sequences of *Pseudomonas* strains used in this study. The tree was constructed with MEGA6 using the Maximum Likelihood Method with 1000 bootstrap replicates. Only bootstrap values above 70% are indicated.

Results from the phylogenetic analyses using *rpoD* and *rpoB* partial sequences of CLP-producing *Pseudomonas* strains show that lokisin-producing strains and arthrofactin-producing strains clustered with putative lokisin and putative arthrofactin producers, respectively. These strains all belong to the *P. koreensis* group and cluster with the type strain *P. koreensis* LMG21318^T^. Our results also suggest that *P. chlororaphis* EA105 and P. *fluorescens* HKI0770 are closely related to the *P. koreensis* group. It is clear that *P. chlororaphis* EA105 does not cluster with the type strain *P. chlororaphis* LMG 1245^T^. Our result also show that the WLIP-producers *Pseudomonas* sp. NSE1 and COW10 and potential WLIP-producing strains NX-1 and PC2 are closely related and also form a cluster with the WLIP-producer RW10S2. The xantholysin producing strain COR51 clustered separately from two other xantholysin producers, BW11M1 and BS011 (Figure 7). The phylogenetic analysis of the housekeeping gene (*rpoD* and *rpoB*) is in agreement with the BLASTp comparison and phylogenetic analysis of the A domains from the CLPs biosynthetic genes.

## Discussion

In this study, we investigated the potential of previously characterized *Pseudomonas* strains and their CLPs (Oni et al., 2019a; Geudens, 2017) to control *M. oryzae* on rice by ISR and direct contact. A summary of the results is given in Table 4. Upon root inoculation *Pseudomonas* strains producing the CLPs N3, lokisin, WLIP and entolysin successfully induced resistance to rice blast, while the xantholysin producer (COR51) did not. In addition, crude CLP extracts obtained from these strains were applied to the rice plant by a soil drench. Lokisin-, WLIP- and entolysin-drenched rice plants successfully induced resistance to the blast disease. However, treatments involving crude extracts of N3 and xantholysin were not effective. These data suggest that the ISR-inducing capacity of *Pseudomonas* strains COR10, COW10 and COR5 is caused by their respective CLPs lokisin, WLIP and entolysin. The CLP xantholysin is not able to trigger ISR against rice blast, while the CLP N3 does not seem to be responsible for the observed ISR in strain COW3. In the case of WLIP, we could further validate the role of this CLP in induced resistance by using the WLIP-producing strain *P. putida* RW10S2 and its WLIP-deficient mutants that were generated previously (Rokni-Zadeh et al., 2012). The wild type strain induced resistance to rice blast to a similar extent as WLIP-producing strains COW10 and NSE1. In contrast, plants treated with all three WLIP mutant strains, were as susceptible to rice blast as the disease control plants.

**Table 4:** Summarizing table showing the potential of *Pseudomonas* strains and their CLPs to control *M. oryzae* on rice by induced systemic resistance and direct effects.

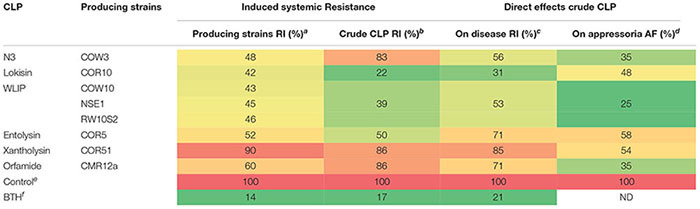

*The color code was given using the conditional formatting tool in Excel with green: very effective and red: no effect. RI, relative infection; AF, appressoria formation; ND, not done. ^*a*^Data from Figure 1 (COW3, COR10, COR5, COR51); Figure 3 (WLIP producers); and Ma et al. (2017) (CMR12a). ^*b*^Data from Figure 2. ^*c*^Data from Figure 4. ^*d*^Number of appressoria relative to DMSO control; values for 25 μM crude extract. Data from Figure 5. ^*e*^Diseased control. ^f^BTH, benzathiadiazol, used as positive control in disease assays*.

Our study presents the first report of the capacity of CLP-producing *Pseudomonas* strains to elicit ISR against rice blast. *Pseudomonas* CLPs have been shown before to be bacterial determinants of ISR. Massetolide, a CLP related to WLIP, was active in tomato against *Phytophthora infestans* (Tran et al., 2007), while orfamide triggered ISR in bean against web blight caused by *Rhizoctonia solani* (Ma et al., 2016b) and in rice against the brown spot pathogen *Cochliobolus miyabeanus* (Ma et al., 2017). The orfamide-producing *P. protegens* strain CHA0, however, was unable to trigger ISR against rice blast (Spence et al., 2014). In addition, we showed that purified orfamide does not elicit ISR against rice blast which confirms results obtained in earlier studies (Ma et al., 2017). The orfamide-producing *Pseudomonas* sp. strain CMR12a however, is able to induce resistance to rice blast, but mutant analysis has revealed that phenazines rather than orfamide are responsible for the observed ISR (Ma et al., 2017). It has been shown that *P. chlororaphis* EA105, an isolate obtained from rice roots in California, can induce systemic resistance against rice blast and triggers jasmonate- and ethylene-dependent defense responses (Spence et al., 2014), but the bacterial determinants involved in ISR were not elucidated. Interestingly, our bioinformatics analysis has revealed that strain EA105 is likely to produce a novel CLP of the amphisin group that is related to lokisin (Supplementary Table S2 and Supplementary Figure S3). It is tempting to speculate that this CLP could play a role in the ISR observed by Spence et al. (2014). In this context it should be noted that *Pseudomonas* sp. GM30, an isolate obtained from *Populus deltoides* roots in the US, is able to trigger ISR against *P. syringae* in *Arabidopsis thaliana* and induces the jasmonate marker *PDF1.2* (Weston et al., 2012). We showed that this strain is a putative arthrofactin-producer (Supplementary Table S2 and Supplementary Figure S3). *P. koreensis* CRS05-R5, another putative arthrofactin producer (Supplementary Table S2 and Supplementary Figure S3), was isolated from rice roots in China (Lin et al., 2016). These results suggest that amphisin group CLPs are produced by *Pseudomonas* isolates associated with the rice rhizosphere and it is worth investigating whether not only lokisin, but also other amphisin group CLPs are ISR elicitors. However, the capacity of CLPs to induce systemic resistance is structure, plant and pathogen dependent. It remains to be investigated whether xantholysin or N3 can act as a resistance inducer in other plant–pathogen combinations.

In addition to ISR, we tested the crude CLP extracts for their capacity to control *M. oryzae* when co-applied with fungal spores on rice leaves. We observed a significant reduction in the number of susceptible blast lesions following treatment of *M. oryzae* VT5M1 spores with 25 μg/ml of each CLP prior to pathogen application on rice leaves, with the exception of xantholysin. Similar observations were observed in a recent study in which up to 50 μM of orfamides could directly reduce rice blast severity after pre-treatment of *M. oryzae* spores prior to foliar inoculation (Ma et al., 2016a). In contrast, 25 μg/ml of crude xantholysin did not protect rice against blast disease. Our findings contradict a previous study which recorded a significant reduction of rice blast severity after spraying rice leaves with 10 and 25 μg/ml of a crude xantholysin extract (obtained from *P. mosselii* BS011) mixed with *M. oryzae* Guy11 spores (Wu et al., 2018). This may be explained by differences in sensitivity toward xantholysin between *M. oryzae* isolates or differences in the bacterial strains used. Wu et al. (2018) observed a strong antagonism between *P. mosselii* BS011 and *M. oryzae* Guy11 in plate assays. We did some preliminary antagonistic tests with *M. oryzae* VT5M1 and *M. oryzae* Guy11 using COR51 and *P. putida* BW11M1, a strain very similar to BS011. Wu et al. (2018) have reported that the xantholysin cluster in BS011 is nearly identical to the cluster in BW11M1 and Figure 7 shows that both strains are also taxonomically closely related. Guy11 is more strongly repressed by COR51 than VT5M1. Moreover, BW11M1 can completely inhibit the growth of both fungi, while this is not the case for COR51 (Supplementary Figure S2). These observations clearly warrant further investigation. In a comparative analysis of the genomes of field isolates of *M. oryzae* it was shown that the field isolates have hundreds of isolate-specific genes, some of which affect virulence (Xue et al., 2012). Thus, possible genetic and physiological differences between *M. oryzae* VT5M1 and *M. oryzae* Guy11 may further account for the different observations in the bioactivity of xantholysin. Besides, it is plausible that different *Pseudomonas* strains produce different amounts of CLPs and in different proportions of congeners. Since the crude CLP extracts used in these studies were not directly quantified, these two studies are not exactly comparable. Future studies using purified CLPs will shed more light on the differences in level of bioactivity among structurally different or similar CLPs.

By means of appressoria, *M. oryzae* penetrates the rice cuticles through turgor pressure generation (Wilson and Talbot, 2009). The direct effect of crude CLPs against spore germination and appressoria formation in *M. oryzae* were tested under *in vitro* conditions. Microscopic examination revealed that there was no difference in spore germination between CLP treatments and the DMSO control. This result is consistent with earlier reports about the effect of orfamide (Ma et al., 2016a) and xantholysin (Wu et al., 2018) on spore germination in *M. oryzae*. In our study, all CLPs tested significantly inhibited appressoria formation at concentrations ranging from 10 to 25 μg/ml. Previous studies showed that purified orfamide A, produced by *P. protegens* CHA0, inhibited appressoria formation at 10 and 50 μM (Ma et al., 2016a). A similar effect on appressoria formation was reported for crude xantholysin produced by *P. mosselii* BS011 when tested against *M. oryzae* at 25 μg/ml (Wu et al., 2018). Another study showed the direct inhibition of appressoria formation in *M. oryzae* by *Pseudomonas* sp. EA105 and *P*. *protegens* CHA0 (Spence et al., 2014). However, the role of CLPs in the inhibition of appressoria by these bacteria was not investigated. As discussed above EA105 likely produces an amphisin-type CLP (this work) while CHA0 is an orfamide producer (Ma et al., 2017). The obstruction of appressoria formation in *M. oryzae* can efficiently lower blast severity on rice plants (Liu et al., 2011; Ma et al., 2016a). Additionally, previous studies showed the inhibition of appressoria formation by orfamides on rice leaves (Ma et al., 2016a) and by mannosylerythritol lipids and Tween 20 on a hydrophobic surface (Yoshida et al., 2015). Yoshida et al. (2015) hypothesized that the inhibition of appressoria formation by *M. oryzae* may be facilitated by changes in surface hydrophobicity due to prior application of surfactants. In our study, we found that *M. oryzae* VT5M1 produces fewer appressoria on microscopic glass slides in comparison with microscopic plastic slides (data not shown). Similar studies showed the capacity of *M. grisea* Guy11 to form appressoria on plastic cover slips but not on glass slides (Skamnioti and Gurr, 2007).

Lokisin was first isolated from *Pseudomonas* strain DSS41 and is a member of the amphisin class of CLPs (Nielsen et al., 2002; Sørensen et al., 2002). In previous studies on the biological activity of CLP-producing strains, whole cells of lokisin-producing *Pseudomonas* sp. DSS41 strain showed antagonistic activity against *Pythium ultimum* and *Rhizoctonia solani* in *in vitro* tests (Nielsen et al., 2002). Also lokisin obtained from *P. koreensis* 2.74 showed biocontrol against *P. ultimum* in a hydroponic tomato cultivation system (Hultberg et al., 2010), while our lokisin-producing COR10 isolate is antagonistic against *P. myriotylum* on cocoyam (Oni et al., 2019a). Till date, the biosynthetic gene cluster of lokisin is yet to be described. Mining of the draft genome of *Pseudomonas* sp. COR10 revealed the presence of three clustered NRPS genes, *lokA*, *lokB* and *lokC*, which are responsible for lokisin biosynthesis in *Pseudomonas* sp. COR10, conforming with the “colinearity rule.” Results of our *in silico* analyses are in congruence with the chemical analysis of lokisin earlier published for *Pseudomonas* sp. COR10 (Oni et al., 2019a). Similar to most CLP gene clusters, the NRPS genes of lokisin are flanked upstream by *nodT*-like and *luxR*-type genes designated *lokT* and *lokR*, respectively. More so, genes encoding the macrolide export protein (MacA) and the ABC transporter (MacB) are located downstream of the *lokABC* genes. In contrast to most CLPs, there is no *luxR* transcriptional regulator downstream of the lokisin NRPS cluster (Figure 6A). This is a rare occurrence in CLP-producing *Pseudomonas* strains. For single CLP-producing strains, the absence of a *luxR* transcriptional regulator downstream of the NRPS cluster is noticeable in the entolysin biosynthetic cluster (Vallet-Gely et al., 2010), in the xantholysin biosynthetic clusters of strain BW11M1 (Li et al., 2013) and the closely related strain BS011 (Wu et al., 2018). In the dual CLP producer *Pseudomonas* sp. CMR12a, a *luxR* regulator was found to be lacking in the downstream position of one of the two CLP gene clusters present (D’aes et al., 2014). Anikasin produced by *P. fluorescens* HKI0770, an isolate obtained from forest soil in Germany (Götze et al., 2017) has the same AAs sequence as lokisin. The predicted D/L-configuration of AAs in COR10 lokisin (to be chemically validated), does also not deviate from the absolute configuration of anikasin. Hence, it cannot be excluded that strains HKI0770 and COR10 produce the same CLP. Both strains appear to be taxonomically related, since *P. fluorescens* HKI0770 also clusters with the *P. koreensis* group (Figure 7). However, significant divergence of the respective NRPS sequences is evident and the genomic context of the biosynthetic gene clusters differs between these strains (Supplementary Table S3 and Supplementary Figure S3).

Unfortunately, we were not able to study the entolysin biosynthetic gene cluster of *Pseudomonas* sp. COR5 and the xantholysin cluster in *Pseudomonas* sp. COR51 in detail because the available genome sequences were very fragmented. We previously showed that *Pseudomonas* sp. COR5 has strong biocontrol activity against *P. myriotylum* on cocoyam (Oni et al., 2019a), while pure entolysin caused hyphal leakage of *P. myriotylum* at concentrations of 25 and 50 μM (Oni et al., 2019a). The NRPS cluster of entolysin has been described for *P. entomophila* L48 (Vallet-Gely et al., 2010). This strain was lethal for insects when ingested at high dose and further showed biocontrol ability against *Pythium ultimum*. However, mutant analysis revealed that its virulence against insects and biocontrol activity were not linked to entolysin production. We found various entolysin producers in the rhizosphere of cocoyam in Cameroon, and these strains, including COR5 belong to the *P. putida* group and are taxonomically related to *P. entomophila* L48. To our knowledge, so far no other entolysin-producing bacteria have been described.

In this study, the role of WLIP in induced resistance to *M. oryzae* was established using the rice rhizosphere isolate, RW10S2, and its WLIP mutants. The anti-*Xanthomonas* activity of rice-rhizospheric *Pseudomonas putida* RW10S2 has been attributed to WLIP production (Rokni-Zadeh et al., 2012). In addition, other WLIP-producing strains were tested including the cocoyam rhizosphere isolate, NSE1. *Pseudomonas* sp. NSE1 showed similar ISR results as RW10S2. Considering the fact that the WLIP biosynthetic machinery of the RW10S2 has been fully elucidated (Rokni-Zadeh et al., 2012), it was interesting to investigate if NSE1 possesses a similar WLIP system as that of RW10S2. We identified the NRPS genes, *wlpA*, *wlpB* and *wlpC* which are responsible for WLIP biosynthesis by *Pseudomonas* sp. NSE1. Similar to RW10S2 (Rokni-Zadeh et al., 2013), *wlpA* and *wlpB-wlpC* are located in distant NRPS clusters (Figure 6B). A similar split organization is found for entolysin (Vallet-Gely *et al.*, 2010), xantholysin (Li et al., 2013), viscosin (De Bruijn and Raaijmakers, 2009), and poaeamide (Zachow et al., 2015). This is contrary to most CLPs which have three biosynthetic genes in the same NRPS cluster, including lokisin (this study), anikasin (Götze et al., 2017), bananamide (Nguyen et al., 2016), arthrofactin (Roongsawang et al., 2003), putisolvin (Dubern et al., 2008), orfamide (Ma et al., 2016a), and gacamide (Jahanshah et al., 2019) among others. WLIP-producing *Pseudomonas* species involved in this study were isolated from diverse ecologies in various countries; rhizosphere of crops (cocoyam and rice) in tropical rain forests of Cameroon (COW10), Nigeria (NSE1), and Sri Lanka (RW10S2). Potential WLIP-producing strains, PC2 and NX-1, isolated from subtropical monsoon climates in China, were obtained from *Pistacia chinensis* and leaf mold, respectively (Supplementary Table S1). This suggests that WLIP is likely found in various ecologies and can probably be named as a core CLP which plays specific roles within the producing strains in their associated ecologies.

The CLPs that we used in our study are structurally diverse and show variation in length of fatty acid tail, number of AAs in the entire oligopeptide length and the size of the macrocyclic lactone ring. At this moment it is not clear which parts of the CLP molecule determine biological activity. All the CLPs tested in this study have a similar 3-hydroxy C10 fatty acid tail except for orfamide A which has a 3-OH C14. However, xantholysin and orfamide, two CLPs that do not elicit ISR against rice blast, differ from the bioactive CLPs (lokisin, WLIP and entolysin) by the presence of eight AA in the peptide ring (Table 5). This relationship is worth exploring in future studies. We recently elucidated the structure of N3 and revealed that it is a novel member of the bananamide group with eight AAs, 6 of which are present in the peptide ring. The full structural elucidation and genome organization of N3 will be published elsewhere.

**Table 5:** Comparison of the oligopeptide sequence and fatty acid moiety of CLPs used in this study.

CLPs	FA^a^	1	2	3^b^	4	5	6	7	8	9	10	11	12	13	14	L:r^c^	References
WLIP	3-OH C10	L-Leu	D-Glu	D-aThr	D-Val	D-Leu	D-Ser	L-Leu	D-Ser	L-Ile						9:7	This study
Orfamide A	3-OH C14	L-Leu	D-Glu	D-aThr	D-Ile	L-Leu	D-Ser	L-Leu	L-Leu	D-Ser	L-Val					10:8	Ma et al., 2016a
Lokisin	3-OH C10	D-Leu	D-Asp	D-aThr	D-Leu	D-Leu	D-Ser	L-Leu	D-Ser	L-Leu	L-Ile	L-Asp				11:9	This study
Entolysin A	3-OH C10	Leu	Glu	Gln	Val	Leu	Gln	Val	Leu	Gln	Ser	Val	Leu	Ser	Ile	14:5	Oni et al., 2019a
Xantholysin A	3-OH C10	Leu	Glu	Gln	Val	Leu	Gln	Ser	Val	Leu	Gln	Leu	Leu	Gln	Ile	14:8	Oni et al., 2019a

*The amino acid configuration of orfamide A is based on the absolute configuration elucidated for strain Pf-5. The amino acid stereochemistry of lokisin (COR10) is predicted from C-domain phylogenetic analysis (Supplementary Figure S3 and Table 4). ^*a*^FA, fatty acid. ^*b*^aThr, *allo*-threonine. ^*c*^L, length of peptide in each CLP; r, number of amino acids involved in the lactone ring of each CLP (residues underlined)*.

Correct taxonomic assignment of CLP-producing *Pseudomonas* species bacteria has been highlighted as a useful clue to identify known and novel CLPs (Oni et al., 2019a). In this study, the taxonomic affiliation of the WLIP-, lokisin-, and xantholysin-producing strains was further established by phylogenetic analysis using partial sequences of *rpoD* and *rpoB* genes. This taxonomic clustering based on housekeeping genes is in congruence with clustering of corresponding A domains and protein sequences for the WLIP and lokisin gene clusters. Our results confirm that of Oni et al. (2019a) and show that a clear taxonomic designation of a CLP-producing *Pseudomonas* strain could serve as a clue for CLP prediction.

## Conclusion

Our study demonstrates the capacity of WLIP-, lokisin-, and entolysin-producing *Pseudomonas* strains to control the rice blast disease caused by *M. oryzae* by ISR and direct effects. The CLPs orfamide and N3 are unable to mount ISR against rice blast but can control the pathogen when co-inoculated together on rice leaves. Additionally, we described the WLIP biosynthetic gene cluster for *Pseudomonas* sp. NSE1 and the lokisin biosynthetic gene cluster for *Pseudomonas* sp. COR10. Our results highlight the biological activity potential of structurally diverse CLPs. Future research is aimed at unraveling the mechanisms by which CLPs trigger defense responses and further studying structure/function relationships in *Pseudomonas* CLPs.

## Data Availability

The datasets generated for this study are available in Genbank, MK534107, MK534106, and MK650230.

## Author Contributions

MH, FO, and OO conceived and designed the research. RD supplied RW10S2 and BW11M1, and their corresponding mutants. OO, HB, and HY conducted the plant experiments. FO isolated *Pseudomonas* sp. NSE1 and prepared DNA materials for genome sequencing. OO performed the rest of the experiments, *in silico* analyses, and analyzed the data. OO and FO wrote the manuscript. MH and RD revised the manuscript and helped in structuring the work. All authors revised the manuscript and approved the final version for submission.

## Conflict of Interest Statement

The authors declare that the research was conducted in the absence of any commercial or financial relationships that could be construed as a potential conflict of interest.

## Footnotes

1 https://blast.ncbi.nlm.nih.gov/Blast.cgi

2 http://www.geneious.com
