# Supplementary material for: Pseudomonas Cyclic Lipopeptides Suppress the Rice Blast Fungus Magnaporthe oryzae by Induced Resistance and Direct Antagonism
Source: Front Plant Sci. 2019 Jul 10;10:901. doi: 10.3389/fpls.2019.00901 (PMC6636606; doi:10.3389/fpls.2019.00901)
Supplement: Supplementary file 1 [file Data_Sheet_1.pdf]

Supplementary Information

CONTENT

Supplementary Figures

Figure S1. Direct effect of crude CLP extracts on spore germination in *M. oryzae*.

Figure S2. In vitro antagonistic test.

Figure S3. Phylogeny-based substrate specificity prediction of lokisin synthetases.

Figure S4. Phylogenetic analysis of condensation domains extracted from lokisin, and from both characterized and putative amphisin group CLPs.

Figure S5. Phylogeny-based substrate specificity prediction of WLIP and putative WLIP synthetases.

Figure S6. Phylogenetic analysis of condensation domains extracted from WLIP- and putative WLIP-producing *Pseudomonas* species.

Supplementary Tables

Table S1. Genomes used in this study

Table S2. Alignment of the amino acid sequence of lokisin produced by *Pseudomonas* sp. COR10 with other (putative) CLPs of the amphisin group.

Table S3. Homology of NRPS enzymes and proteins encoded by the flanking regions identified from the lokisin-producing *Pseudomonas* sp. COR10 and related strains.

Table S4. WLIP synthetases and proteins encoded by the flanking region identified from *Pseudomonas* sp. NSE1 and related strains.

Table S5. Alignment of amino acids sequence of WLIP produced by *Pseudomonas* sp. NSE1 and other strains

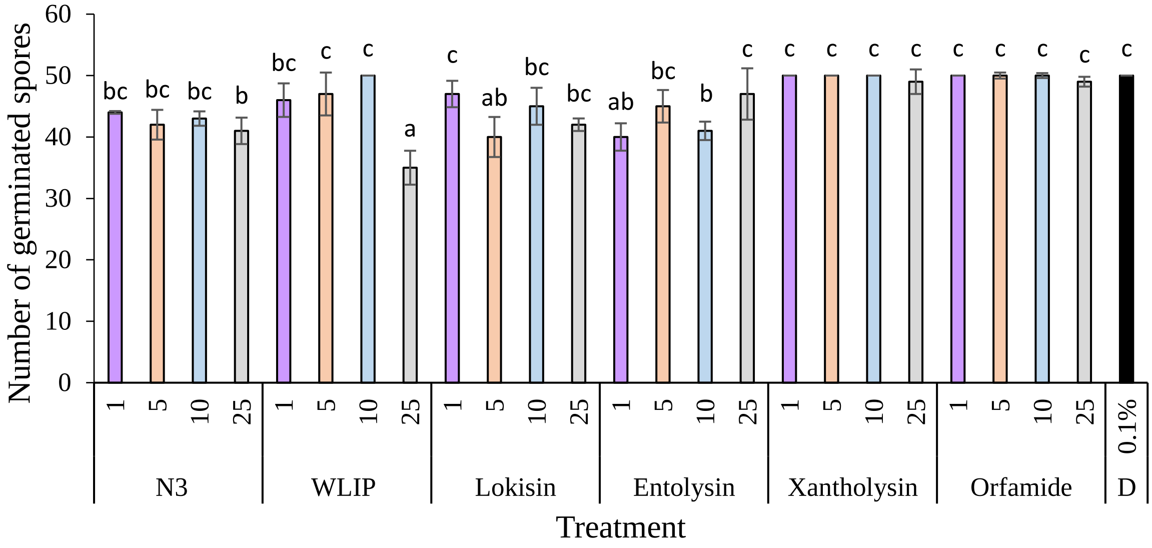

Figure S1. Direct effect of crude CLP extracts on spore germination in *M. oryzae*. Crude extracts were tested at concentrations ranging from 1 to 25 µg/ml. Different letters indicate statistically significant differences among the different treatments (Anova followed by a Tukey's test; α=0.05). Standard deviations are shown (n=5). D: dimethyl sulfoxide (DMSO) control.

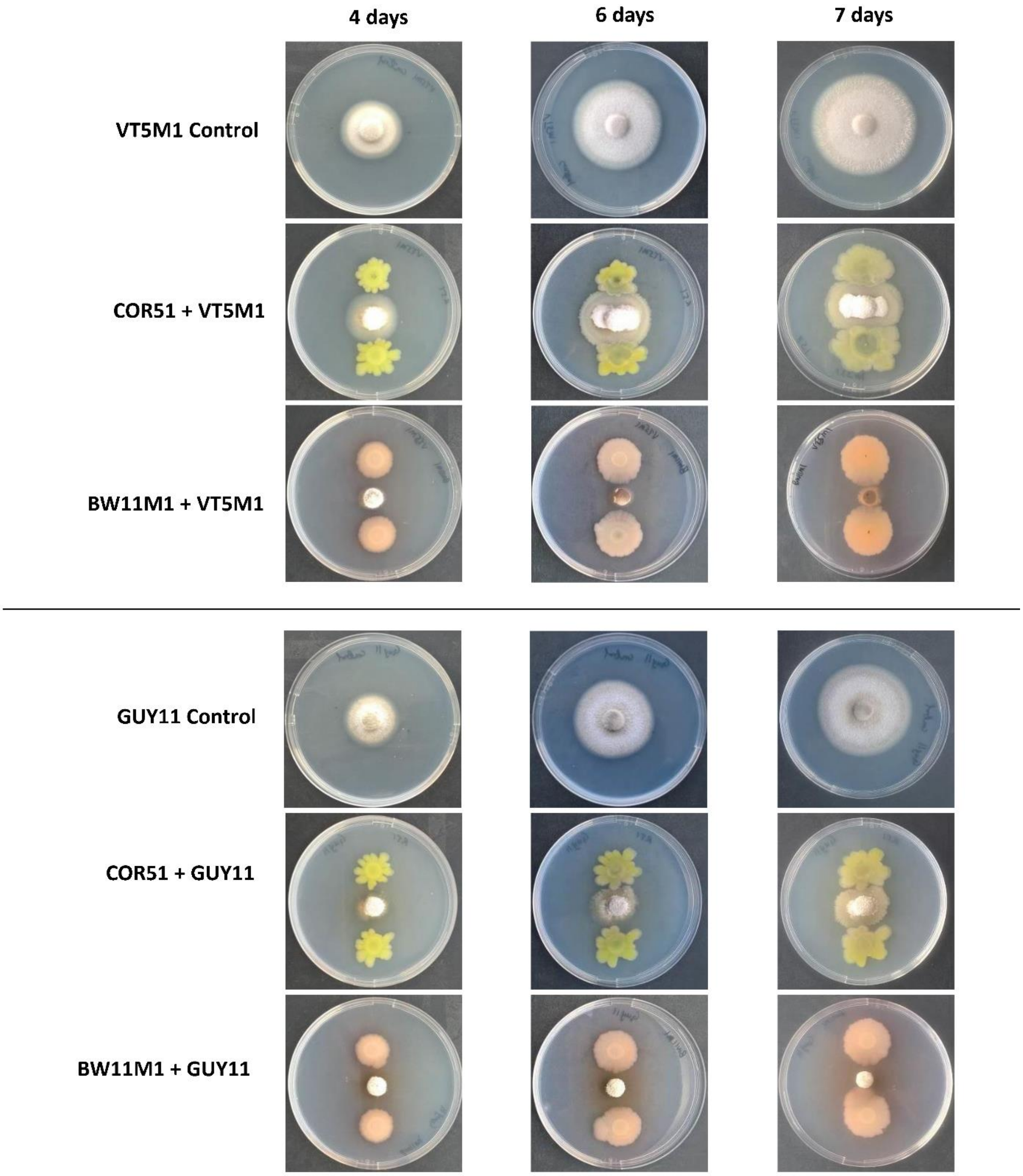

Figure S2. In vitro antagonistic test. Co-culturing of xantholysin producing strains COR51, and BW11M1 with *M. oryzae* VT5M1 and M. oryzae Guy11. Representative pictures were taken 4, 6 and 7 days, respectively, after the pathogen was added to the center of the Petri dish. The experiment was carried out with 3 repetitions and was repeated in time.

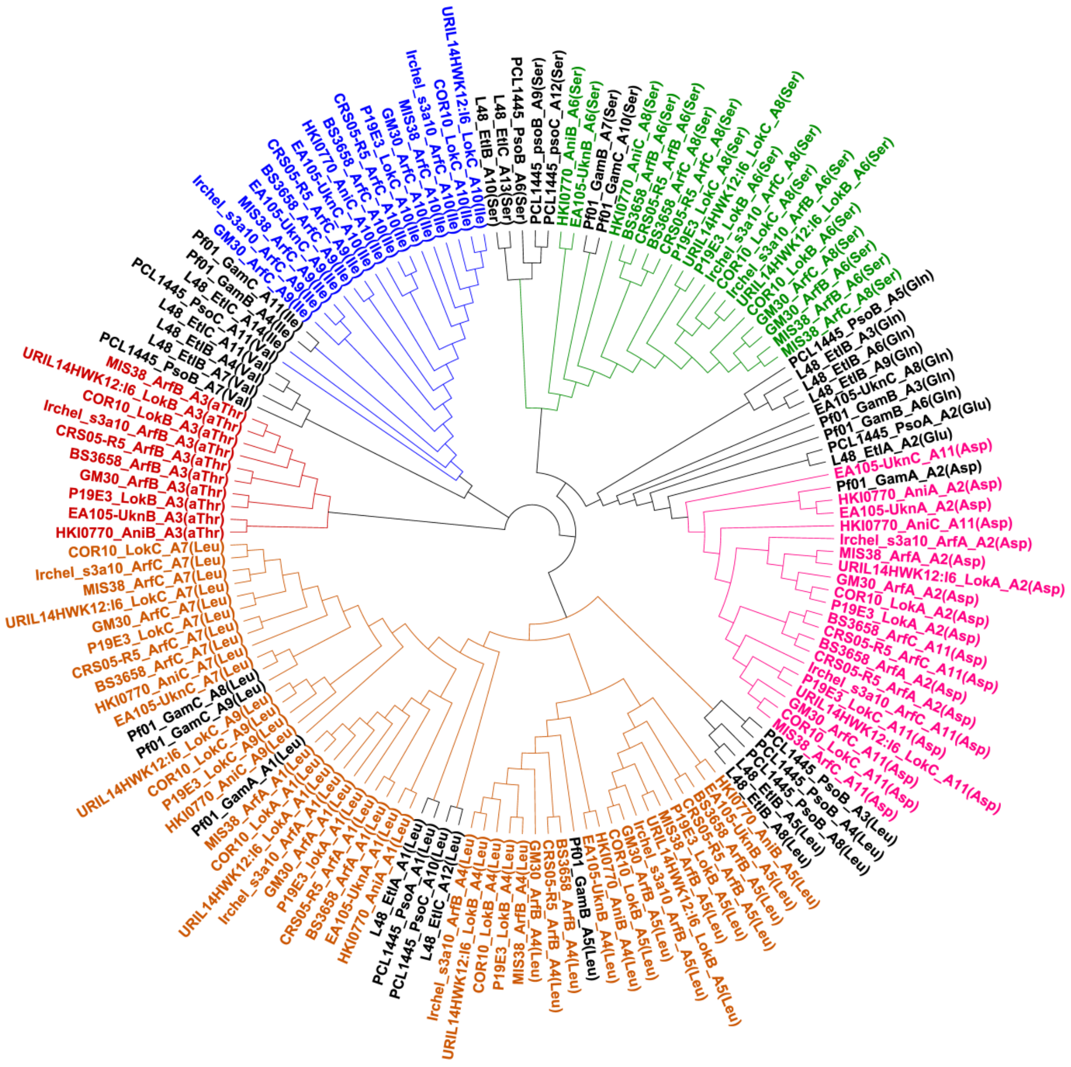

Figure S3. Phylogeny-based substrate specificity prediction of lokisin synthetases.

Cladogram of neighbor joining tree inferred from amino acid sequence alignment of adenylation (A) domains extracted from already characterized and putative *Pseudomonas* CLP NRPSs in the amphisin group. Lipopeptide-specific codes are: Etl (entolysin, *P. entomophila* L48); Pso (putisolvin, *P. putida* PCL1445); Gam (gacamide, *P. fluorescens* Pf01); Arf (arthrofactin, *P. fluorescens* MIS38); Lok (lokisin, *Pseudomonas* sp. COR10); Ani (anikasin, *P. fluorescens* HKI0770); Arf (putative arthrofactin, *P. koreensis* BS3658); Lok (putative lokisin, *P. koreensis* P19E3); Arf (putative arthrofactin, *P. koreensis* CRS05-R5); Ukn (putative new member of the amphisin group, *P. chlororaphis* EA105); Arf (putative arthrofactin, *Pseudomonas* sp. GM30); Lok (putative lokisin, *Pseudomonas* sp. URIL14HWK12:I6); and Arf (putative arthrofactin, *Pseudomonas* sp. Irchel_s3a10). For each domain the substrate specificity (known or inferred) is indicated in parentheses using the standard amino acid three-letter code. The non-protein amino acid is annotated as follows: *allo*-threonine (aThr). Clusters comprising lokisin domains with different specifities are highlighted in different colors.

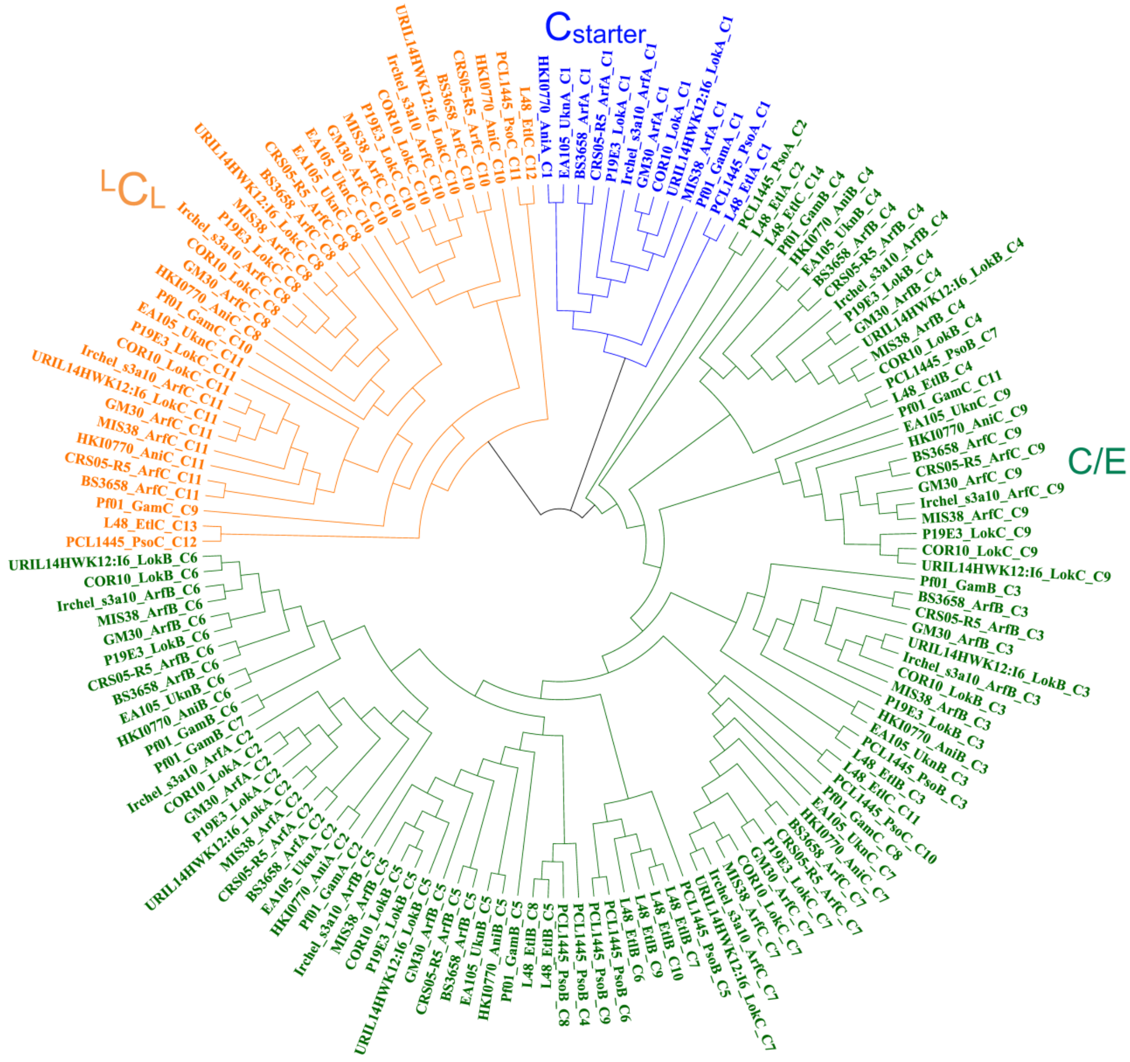

Figure S4. Phylogenetic analysis of condensation domains from lokisin, and other characterized and putative amphisin group CLPs.

Cladogram of neighbor joining tree inferred from amino acid sequence alignment of condensation (C) domains extracted from already characterized and putative *Pseudomonas* CLP NRPSs belonging to the amphisin group. Lipopeptide-specific codes are: Etl (entolysin, *P. entomophila* L48); Pso (putisolvin, *P. putida* PCL1445); Gam (gacamide, *P. fluorescens* Pf01); Arf (arthrofactin, *P. fluorescens* MIS38); Lok (lokisin, *Pseudomonas* sp. COR10); Ani (anikasin, *P. fluorescens* HKI0770); Arf (putative arthrofactin, *P. koreensis* BS3658); Lok (putative lokisin, *P. koreensis* P19E3); Arf (putative arthrofactin, *P. koreensis* CRS05-R5); Ukn (putative new member of the amphisin group, *P. chlororaphis* EA105); Arf (putative arthrofactin, *Pseudomonas* sp. GM30); Lok (putative lokisin, *Pseudomonas* sp. URIL14HWK12:I6); and Arf (putative arthrofactin, *Pseudomonas* sp. Irchel_s3a10).

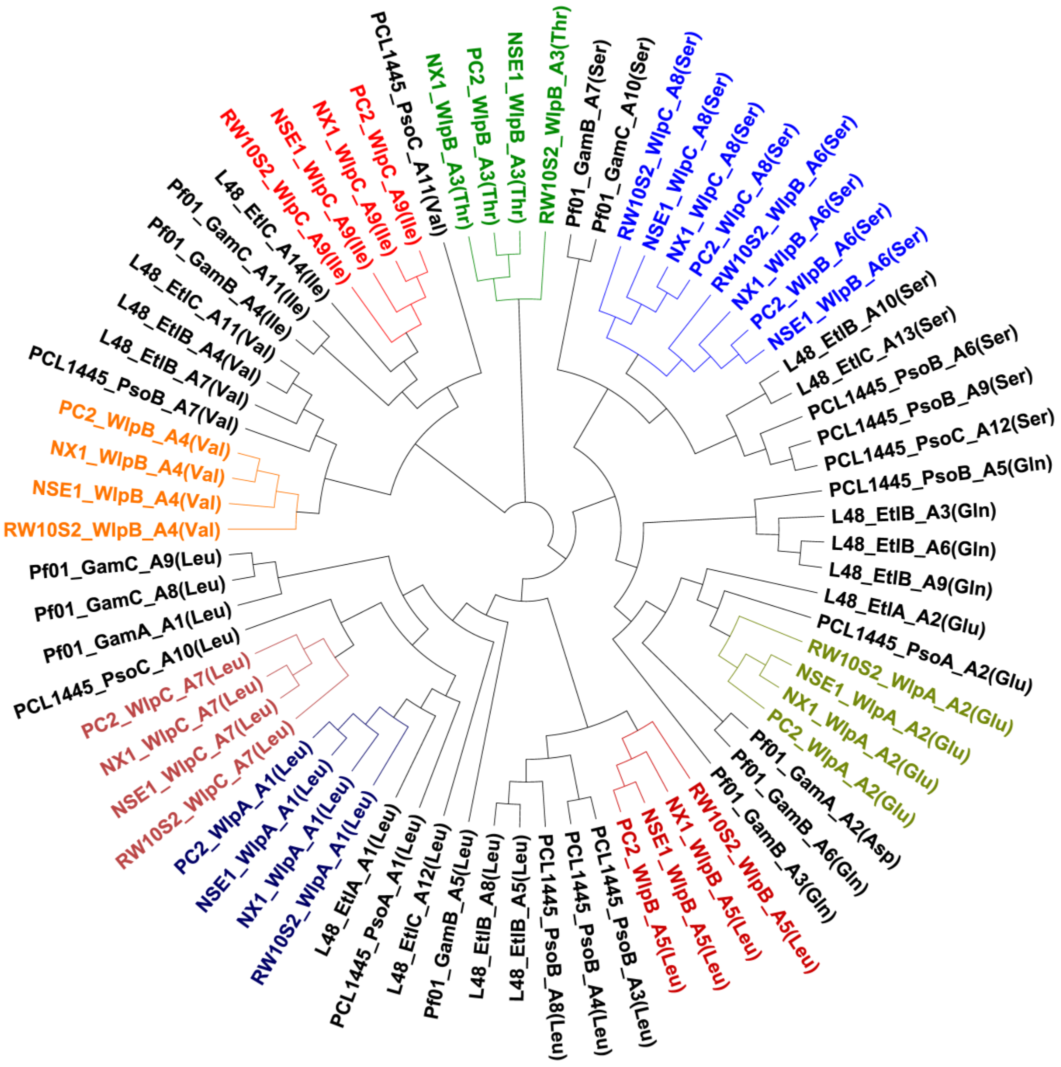

Figure S5. Phylogeny-based substrate specificity prediction of WLIP synthetases. Cladogram of neighbor joining tree inferred from amino acid sequence alignment of adenylation (A) domains extracted from already characterized and putative *Pseudomonas* CLP NRPSs for the biosynthesis of WLIP. Lipopeptide-specific codes are: Etl (entolysin, *P. entomophila* L48); Pso (putisolvin, *P. putida* PCL1445); Gam (gacamide, *P. fluorescens* Pf01); Wlp (WLIP, *P. putida* RW10S2, and *Pseudomonas* sp. NSE1); Wlp (putative WLIP, *P. putida* PC2 and NX1). For each domain the substrate specificity is indicated in parentheses using the standard amino acid three-letter code. Clusters comprising WLIP domains are highlighted in different colors.

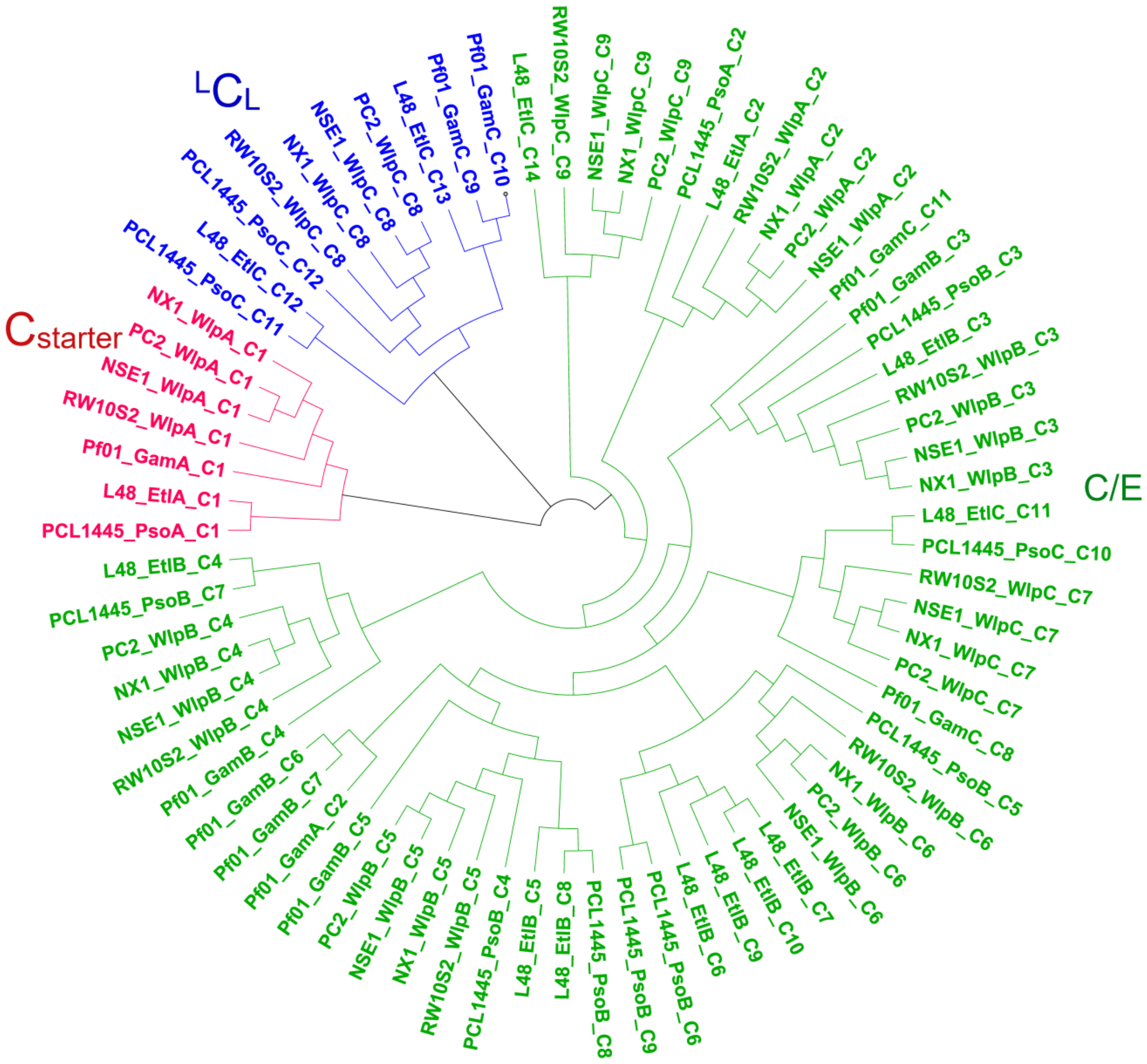

Figure S6. Phylogenetic analysis of condensation domains from WLIP- and putative WLIP-producing *Pseudomonas* species.

Cladogram of neighbor joining tree inferred from amino acid sequence alignment of condensation (C) domains extracted from already characterized and putative WLIP-producing strains. Lipopeptide-specific codes are: Etl (entolysin, *P. entomophila* L48); Pso (putisolvin, *P. putida* PCL1445); Gam (gacamide, *P. fluorescens* Pf01); Wlp (WLIP, *P. putida* RW10S2, and *Pseudomonas* sp. NSE1); Wlp (putative WLIP, *P. putida* PC2 and NX1).

Table S1. *Pseudomonas* genomes/sequences retrieved from GenBank

Strain	Origin	CLP	Accession	Reference
P. koreensis BS3658	-	ND	NZ_LT629687	Unpublished
P. chlororaphis EA105	Rice roots, California, USA	ND	JSFK00000000	McCully et al., 2014; Spence et al. 2014
Pseudomonas sp. URIL14HWK12:I6	-	ND	NZ_AZVL01000000	Unpublished
P. koreensis P19E3	Leaf of *Origanum majorana*, Switzerland	ND	CP027477, CP027481	Schmid et al., 2018
P. fluorescens MIS38	Oil spill soil, Japan	arthrofactin	AB107223.1, AB286215.1	Lange et al., 2012
Pseudomonas sp. Irchel_s3a10	Soil, Switzerland	ND	GCA_900187525.1	Butaite et al., 2017
Pseudomonas sp. GM30	*Populus deltoides* roots, Tennessee, USA	ND	GCF_000282275.2	Weston et al., 2012
P. koreensis CRS05-R5	Rice rhizosphere, China	ND	CP015852	Lin et al., 2016
P. fluorescens HKI0770	Forest soil sample, Germany	anikasin	LVEJ01000000	Götze et al. 2017
P. putida RW10S2	Rice rhizosphere, Sri Lanka	WLIP	JN982332.1, JN982333.1	Rokni-Zadeh et al., 2012
P. putida PC2	*Pistacia chinensis*, China	ND	CP011789.1	Song et al., 2017
P. putida NX-1	Leaf mold, China	ND	CP030750.1	Xu et al., 2018
**Sequence**		**Housekeeping genes accession**		
		rpoB	*rpoD*	
P. fluorescens LMG 5167^T	-	AJ748158	D86033	Ait Tayeb *et al.*, 2005
P. chlororaphis subsp. aureofaciens LMG 1245^T	-	AJ717426.1	FN554453.1	Ait Tayeb et al., 2005
P. koreensis LMG 21318^T	-	FN554737	FN554476	Mulet *et al.*, 2010
P. putida LMG 2257^T	-	AJ717474	AB039581	Ramette *et al.*, 2011
P. aeruginosa LMG 1242^T	-	AJ717442	AB039611	Yamamoto et al., 2000; Ait Tayeb et al., 2005

*ND, Not determined

Table S2. Alignment of the amino acid sequence of lokisin produced by *Pseudomonas* sp. COR10 with other (putative) CLPs of the amphisin group. The lipopeptide structures of arthrofactin (MIS38), anikasin (KHI0770), and lokisin (DSS41, COR10) have been chemically elucidated. The amino acid stereochemistry was determined for arthrofactin (complete), anikasin (complete) and DSS41 lokisin (partial). The other structures are predictions based on phylogenetic analysis of adenylation domains (see Figure S1). Their probable amino acid configurations are predicted by phylogenetic analysis of C-domains (Fig. S2).

Pseudomonas sp.	CLP	FA	A1	A2	A3	A4	A5	A6	A7	A8	A9	A10	A11
COR10	Lokisin	3-OH C10	D-Leu	D-Asp	D-aThr	D-Leu	D-Leu	D-Ser	L-Leu	D-Ser	L-Leu	L-Ile	Asp
DSS41	Lokisin	3-OH C10	Leu	Asp	D-aThr	Leu	Leu	D-Ser	Leu	D-Ser	Leu	L-Ile	Asp
URIL14HWK12:I6	Putative lokisin	FA	D-Leu	D-Asp	D-aThr	D-Leu	D-Leu	D-Ser	L-Leu	D-Ser	L-Leu	L-Ile	Asp
P19E3	Putative lokisin	FA	D-Leu	D-Asp	D-aThr	D-Leu	D-Leu	D-Ser	L-Leu	D-Ser	L-Leu	L-Ile	Asp
MIS38	Arthrofactin	C10 (3-OH)	D-Leu	D-Asp	D-aThr	D-Leu	D-Leu	D-Ser	L-Leu	D-Ser	L-Ile	L-Ile	L-Asp
Irchel_s3a10	Putative arthrofactin	FA	D-Leu	D-Asp	D-aThr	D-Leu	D-Leu	D-Ser	L-Leu	D-Ser	L-Ile	L-Ile	Asp
GM30	Putative arthrofactin	FA	D-Leu	D-Asp	D-aThr	D-Leu	D-Leu	D-Ser	L-Leu	D-Ser	L-Ile	L-Ile	Asp
CRS05-R5	Putative arthrofactin	FA	D-Leu	D-Asp	D-aThr	D-Leu	D-Leu	D-Ser	L-Leu	D-Ser	L-Ile	L-Ile	Asp
BS3658	Putative arthrofactin	FA	D-Leu	D-Asp	D-aThr	D-Leu	D-Leu	D-Ser	L-Leu	D-Ser	L-Ile	L-Ile	Asp
HKI0770	Anikasin	C10 (3-OH)	D-Leu	D-Asp	D-aThr	D-Leu	D-Leu	D-Ser	L-Leu	D-Ser	L-Leu	L-Ile	L-Asp
EA105	New CLP	FA	D-Leu	D-Asp	D-aThr	D-Leu	D-Leu	D-Ser	L-Leu	D-Gln	L-Ile	L-Ile	Asp

Table S3. Homology of NRPS enzymes and proteins encoded by the flanking regions identified from the lokisin-producing *Pseudomonas* sp. COR10 and related strains. Identity level of protein sequences were compared to those of *Pseudomonas* sp. COR10 by BLASTp search. The gene numbers in the first column refer to the gene cluster in Figure 6A.

Gene	NRPS and flanking proteins	URIL14HWK12:I6	P19E3	Irchel_s3a10	GM30	MIS38	BS3658	CRS05-R5	HKI0770	EA105
1	ABC-type nitrate/sulfonate/bicarbonate transport systems, periplasmic components	99% WP_027613132.1	-	99% WP_095139611.1	97% WP_007965827.1	NA	-	-	-	95%
2	Hypothetical protein	89% WP_027613131.1	82%	83% WP_095139609.1	90% WP_007965822.1	NA	77%	77%	28%	66%
3	Copper metallochaperone, bacterial analog of Cox17 protein	-	96%	99% WP_095139608.1	99% WP_007965820.1	NA	94%	94%	89%	86%
4	Cytochrome oxidase biogenesis protein Sco1/ SenC/PrrC, putative copper metallochaperone	99% WP_027613129.1	97%	98% WP_102357144.1	89% WP_064595539.1	NA	99%	99%	90%	89%
lokT	RND efflux system, outer membrane lipoprotein	98% WP_049827914.1	95%	96% WP_095139607.1	97% WP_007965818.1	NA	94%	94%	90%	90%
lokR	LuxR family transcriptional regulator	99% WP_027613127.1	98%	98% WP_095139606.1	99% WP_007965817.1	NA	98%	98%	90%	88%
lokA	Non-ribosomal peptide synthetase	97% WP_027613126.1	94%	95% WP_095139605.1	96% WP_024164422.1	97% BAC67534.2	90%	90%	76%	77%
lokB	Non-ribosomal peptide synthetase	97% WP_027613125.1	96%	97% WP_095139604.1	95% WP_007965814.1	97% BAC67535.1	93%	92%	83%	83%
lokC	Non-ribosomal peptide synthetase	97% WP_027613124.1	95%	90% WP_095139603.1	90% WP_024164421.1	90% BAC67536.1	86%	86%	88%	77%
macA	Macrolide efflux protein MacA	99% WP_027613123.1	99%	98% WP_095139602.1	99% WP_007969710.1		97%	97%	91%	91%
macB	Macrolide efflux protein MacB	99% WP_098968302.1	99%	98% WP_095139601.1	99% WP_007969709.1	99% BAF40423.1	96%	96%	92%	91%
5	Hypothetical protein	-	-	-	-	-	-	-	-	-
6	Tryptophan synthase beta chain like	96% WP_027613717.1	94%	98% WP_095139600.1	95% WP_024164504.1	94% BAF40424.1	-	-	-	-
7	Hypothetical protein	93% WP_027613716.1	-	93% WP_095135582.1	91% WP_007966403.1	93% BAF40425.1	-	-	-	-
8	Hypothetical protein	78% WP_049827959.1	-	85% WP_095135584.1	-	NA	-	-	-	-

Table S4. WLIP synthetases and proteins encoded by the flanking region identified from *Pseudomonas* sp. NSE1 and related strains. Identity level of protein sequences were compared by BLASTp search. The gene numbers in the first column refer to Figure 6B.

		Pseudomonas **sp.**		
**Gene**	**WLIP synthetases and flanking proteins**	**RW10S2**	**NX-1**	**PC2**
1	TraX family protein	95%	100%	100%
2	Transcriptional regulator, LysR family	98%	99%	100%
3	Probable monooxygenase	98%	99%	99%
		AFJ23816.1	AXA25193.1	ANC03467.1
wlpT	RND efflux system, outer membrane	94%	99%	99%
	lipoprotein	AFJ23817.1	AXA25192.1	ANC03466.1
wlpR	LuxR family transcriptional regulator	94%	99%	100%
		AFJ23818.1	AXA25191.1	ANC03465.1
wlpA	Non-ribosomal peptide synthetase	95%	99%	99%
		AFJ23819.1	AXA25190.1	ANC03464.1
4	Transporter, LysE family	94%	100%	100%
5	Integral membrane protein	95%	100%	100%
6	Hypothetical protein	92%	100%	100%
7	Pli0014 protein	-	100%	100%
8	3-demethylubiquinone-9 3-methyltransferase	-	100%	100%
9	Outer membrane ferripyoverdine receptor	-	99%	99%
1	PvdE, pyoverdine ABC export system	-	100%	100%
2	L-ornithine 5-monooxygenase, PvdA	-	100%	100%
2	Hypothetical protein	-	100%	100%
4	PvdO, Pyoverdine responsive serine/threonine kinase	-	100%	100%
5	Pyoverdin biosynthesis protein, PvdN	-	100%	100%
6	Putative dipeptidase, pyoverdin biosynthesis PvdM	-	100%	100%
7	Alkanesulfonates-binding protein	-	100%	100%
8	Acyl-CoA dehydrogenase	99%	100%	99%
9	FMN reductase	99%	99%	100%
wlpB	Non-ribosomal peptide synthetase	96%	99%	99%
		AFJ23825.1	AXA25123.1	ANC03402.1
wlpC	Non-ribosomal peptide synthetase	94%	99%	99%
		AFJ23826.1	AXA25122.1	ANC03401.1
wlpD	Macrolide efflux protein MacA	99%	100%	100%
		AFJ23827.1	AXA25121.1	ANC03400.1
wlpE	Macrolide efflux protein MacB	99%	100%	100%
		AFJ23828.1	AXA25120.1	ANC03399.1
10	Oligopeptide ABC transporter, OppA	70%	99%	99%
11	Oligopeptide ABC transporter, OppA	94%	97%	97%
12	LuxR family transcriptional regulator	96%	100%	99%
13	Inositol-1-monophosphatase	97%	100%	100%
14	Peptidyl-prolyl cis-trans isomerase PpiC	100%	100%	100%
15	Acetoacetyl-CoA synthetase	95%	99%	100%

References: NX-1 (Xu et al., 2018); PC2 (Song et al., 2017); RW10S2 (Rokni-Zadeh et al., 2012)

Table S5. Alignment of amino acids sequence of WLIP produced by *Pseudomonas* sp. NSE1 and other strains

**Strains**	**FA**	**A1**	**A2**	**A3**	**A4**	**A5**	**A6**	**A7**	**A8**	**A9**
Pseudomonas sp. NSE1	3-OH C10	L-Leu	D-Glu	D-Thr	D-Val	D-Leu	D-Ser	L-Leu	D-Ser	L-Ile
Pseudomonas putida RW10S2	3-OH C10	L-Leu	D-Glu	D-Thr	D-Val	D-Leu	D-Ser	L-Leu	D-Ser	L-Ile
Pseudomonas putida NX-1	ND	L-Leu	D-Glu	D-Thr	D-Val	D-Leu	D-Ser	L-Leu	D-Ser	L-Ile
Pseudomonas putida PC2	ND	L-Leu	D-Glu	D-Thr	D-Val	D-Leu	D-Ser	L-Leu	D-Ser	L-Ile

***FA, Fatty acid
ND, Not determined
